# A Review of the Pharmacological Activities and Recent Synthetic Advances of γ-Butyrolactones

**DOI:** 10.3390/ijms22052769

**Published:** 2021-03-09

**Authors:** Joonseong Hur, Jaebong Jang, Jaehoon Sim

**Affiliations:** 1 Natural Products Research Institute, Korea Institute of Science and Technology (KIST), 679 Saimdang-ro, Gangneung 25451, Korea; hjs1120@snu.ac.kr; 2 College of Pharmacy, Korea University, Sejong 30019, Korea; 3 College of Pharmacy, Chungnam National University, Daejeon 34134, Korea

**Keywords:** *γ*-butyrolactone, pharmacological activities, lactone synthesis, lactonization, recent advances

## Abstract

*γ*-Butyrolactone, a five-membered lactone moiety, is one of the privileged structures of diverse natural products and biologically active small molecules. Because of their broad spectrum of biological and pharmacological activities, synthetic methods for *γ*-butyrolactones have received significant attention from synthetic and medicinal chemists for decades. Recently, new developments and improvements in traditional methods have been reported by considering synthetic efficiency, feasibility, and green chemistry. In this review, the pharmacological activities of natural and synthetic *γ*-butyrolactones are described, including their structures and bioassay methods. Mainly, we summarize recent advances, occurring during the past decade, in the construction of *γ*-butyrolactone classified based on the bond formation in *γ*-butyrolactone between (i) C5-O1 bond, (ii) C4-C5 and C2-O1 bonds, (iii) C3-C4 and C2-O1 bonds, (iv) C3-C4 and C5-O1 bonds, (v) C2-C3 and C2-O1 bonds, (vi) C3-C4 bond, and (vii) C2-O1 bond. In addition, the application to the total synthesis of natural products bearing *γ*-butyrolactone scaffolds is described.

## 1. Introduction

*γ*-Butyrolactone, a five-membered heterocycle containing ester functionality, has been broadly studied in the drug discovery field since it is one of the privileged structures of biologically active small molecules. Several *γ*-butyrolactone-containing drugs have been FDA-approved and used in clinic for diverse purposes such as diuretics, anticancer agents, contraceptive drugs, treatment of heart disease, and anti-glaucoma agents. *γ*-Butyrolactone moiety is also found in a variety of biologically active experimental drugs [1,2,3,4] and synthetic intermediates [5,6,7,8,9,10]. Moreover, numerous natural products, showing diverse biological activities, have *γ*-butyrolactone moiety. 

The most universal synthetic method for *γ*-butyrolactone is intramolecular esterification, which can be readily utilized with substrates bearing *γ*-hydroxybutanoic acid functionality. However, diverse synthetic methodologies have been developed based on the discovery of biologically active synthetic or natural lactone drugs. Consequently, there have been many efforts to develop efficient synthetic methods to construct *γ*-butyrolactone, and several focused reviews have been published [11,12,13,14]. For example, Taylor and colleagues summarized new synthetic approaches for *α*-methylene-*γ*-butyrolactones [12] and Marstral, Feringa and colleagues reviewed the catalytic asymmetric synthesis of *γ*-butyrolactone [13]. 

**Figure 1:**
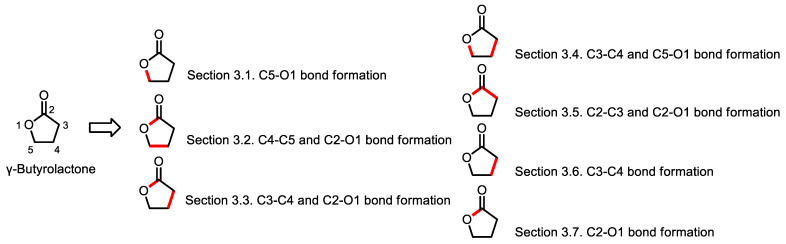
Bond disconnections for the synthesis of *γ*-butyrolactones.

In this review, we first prepare a brief introduction of biologically active *γ*-butyrolactones including eight FDA-approved drugs (Table 1) and various natural and synthetic *γ*-butyrolactones that have broad biological activities such as anticancer, anti-inflammatory, antibiotic, antifungal, antioxidant activities as well as immunosuppressive, neuroprotective, and hypoglycemic activities (Table 2). Additionally, we summarize synthetic methodologies for the construction of *γ*-butyrolactone reported from 2010 to 2020, which are depicted in seven main sections based on the sites of bond formation (Figure 1). Each section is further divided into subsections according to the type of reaction and contains a description focused on the reaction mechanism. Additionally, applications of the reaction to the synthesis of complex molecules are included to demonstrate the synthetic utility of the reactions. The synthetic methodology has been continuously improving over the past decade. Therefore, this review will provide an update of recent work in the development of synthetic methods for the construction of *γ*-butyrolactones. 

## 2. Pharmacological Activities of *γ*-Butyrolactones

### 2.1. Approved Drugs 

Several *γ*-butyrolactone-containing drugs have been FDA-approved and used in clinics for diverse purposes (Table 1). Pilocarpine, isolated from *Pilocarpus microphyllus*, is used to treat xerostomia and reduce eye pressure. (Entry 1) [15]. Pilocarpine is also widely applied to pharmacological research as a control cholinergic agonist. *γ*-Butyrolactone moiety was employed in a steroid skeleton at the C-17 position to develop steroidal aldosterone antagonists (Entry 2 and 3). Spironolactone and eplerenone are common medications for cardiovascular diseases such as high blood pressure and heart failure [16,17]. Drospirenone, structurally similar with spironolactone, is used to prevent pregnancy as a progesterone agonist. (Entry 4) [18]. Podophyllotoxin, a natural DNA topoisomerase inhibitor from *Podophyllum peltatum*, is treated to kill genital warts (Entry 5) [19]. Two semisynthetic derivatives of podophyllotoxin, etoposide, and teniposide, were approved as anticancer agents used for lymphoma, leukemia, and various solid tumors (Entry 6 and 7) [20,21]. Vorapaxar, a derivative of himbacine, is a first-in-class protease-activated receptor-1 (PAR-1) antagonist (Entry 8) [22]. By inhibiting PAR-1, vorapaxar reduces thrombotic cardiovascular events and the risk of myocardial infarction. Now, several *γ*-butyrolactone-containing drug candidates have been investigated in clinical studies for the treatment of heart disease, rheumatoid arthritis, and infectious disease.

### 2.2. Biologically Active γ-Butyrolactones 

#### 2.2.1. Anti-Inflammation

Diverse butyrolactones have been studied to evaluate anti-inflammatory activities (Entry 1–9 in Table 2). Some of these butyrolactones modulate the NF-κB signaling pathway such as a santonine-derived butyrolactone that showed anti-inflammatory activity through the inhibition of the ubiquitin-conjugating enzyme, UbcH5c (Entry 1 in Table 2) [23,24]. This anti-inflammatory activity was maintained in vivo using Freund’s adjuvant arthritis rat model. A novel phthalide-based butyrolactone (Entry 2) [25,26] and two natural products—calcaratarin D (Entry 3) [27] and a sesquiterpene lactone (Entry 4) [28]—were also reported to inhibit activity of the NF-κB signaling pathway and showed anti-inflammatory activity. Among them, the in vivo activity of the first butyrolactone (Entry 2) was evaluated against the adjuvant arthritis rat. Moreover, a biyouyanagin derivative attached to adenine (Entry 5) [29] and arctiidilactone (Entry 6) [30] showed anti-inflammatory activity through the inhibition of LPS-induced cytokine production or LPS-induced NO production, respectively. A COX-2 inhibitor (Entry 7), which is an indole-based *γ*-butyrolactone, was reported to have shown anti-inflammatory activity with an IC_50_ value of <0.001 μM [31]. CD10847 (Entry 8) [32] and cinatrin C3 (Entry 9) [33] exhibited potent anti-inflammatory activities via inhibition of caspase-1 or phospholipase A1, respectively. 

#### 2.2.2. Anticancer

The development of anticancer drugs is one of the long-term goals in the drug development field. Diverse natural and synthetic butyrolactones have been evaluated for their cytotoxic activities against various cancer cell lines. Protelichesterinic acid (Entry 10), a metabolite isolated from *Antarctic lichens*, showed cytotoxicity against HCT-116 cells with an IC_50_ value of 34.3 μM [34]. P. K. Roy and colleagues isolated one of the cembrane-type butyrolactones (Entry 11) from the soft coral, *Lobophytum*, which displayed a strong cytotoxic activity against RAW 264.7 cells [35]. Sasaki and colleagues evaluated the AKT inhibitory activities of lactoquinomycin (Entry 12) [36,37], kalafungin (Entry 13) [36,38], and frenolicin B (Entry 14) [36,39], classified as pyranonaphthoquinone lactones, which were originally reported as antibiotics. These butyrolactones exhibited strong AKT inhibitory activities with IC_50_ values of 0.149 μM~0.313 μM as well as cytotoxic activities with IC_50_ values of 0.05 μM~0.07 μM in MDA468 cells. A cytotoxicity of synthetic butyrolactones has been reported as well. Lee and colleagues synthesized an adenine-linked butyrolactone (Entry 15) which exhibited a cytotoxicity with an ED_50_ value of 0.3 μg/mL in L1210 cells [40]. Another example of synthetic butyrolactone, reported by Huth and colleagues, displayed strong HSP90 inhibitory activity (Ki = 1.9 μM) which could result in the development of anti-cancer agent (Entry 16) [41].

#### 2.2.3. Antibiotic

Many *γ*-butyrolactone-containing small molecules have been studied in the development of antibiotics. Lactivicin (Entry 17) [42,43], produced by two strains of bacteria, and one bicyclic butyrolacone (Entry 18) [44] showed strong inhibition of β-lactamase with IC_50_ values of 2.4 μg/mL and 15 μg/mL, respectively. Moreover, various synthetic *γ*-butyrolactones exhibited potent antibacterial activities. For example, a synthetic *α*-amino-*γ*-lactone ketolide (Entry 19) showed excellent antibacterial activity against erythromycin-susceptible *Streptococus pyogenes* [45]. Additionally, hydrazonothiazolyl derivative (Entry 20) [46], *β*-cyclocitral derivative (Entry 21) [47], and *α*-methylene-*γ*-butyrolactone (Entry 22) [48] displayed potent antibacterial activities and a synthetic *β*-aryl-*δ*-iodo-*γ*-butyrolactone (Entry 23) exhibited bactericidal activity against *Proteus mirabilis* [49,50]. 

#### 2.2.4. Antifungal

Researchers found that α-methylene-*γ*-butyrolactone ring is a natural pharmacophore for antifungal natural products (Entry 24) [51]. Various synthetic *α*-methylene-*γ*-butyrolactone analogues were synthesized and evaluated as potent antifungal agents. Feng’s groups and Xing’s groups found that *α*-methylene-*γ*-butyrolactones bearing aromatic moiety at *γ*-position exhibited antifungal activity against *Colletotrichum lagenarium* (Entry 25,26) [52,53]. Höfle and colleagues isolated complex *γ*-butyrolactone natural product, leupyrrin A1 (Entry 27) from *Sorangium cellulosum* and found its potent antifungal activity [54]. Menche and colleagues reported the first total synthesis of leupyrrin A1 and SAR studies of leupyrrin analogues as potent antifungal agents [55,56]. 

#### 2.2.5. Immunosuppressive

Two synthetic *γ*-butyrolactones and two natural products were reported to show immunosuppressive activities. Yang and colleagues found that benzene-fused *γ*-butyrolactones (Entry 28) demonstrate highly efficacious immunosuppressive properties [57]. A sesquiterpene lactone, isolated from *Artemisia argyi* (Entry 29), also exhibited potent immunosuppressive activity, which was assessed via inhibitory effect on the proliferation of T lymphocytes [58]. A santonin derivative (Entry 30) reported by Chinthakindi and colleagues is another example of the immunosuppressant evaluated by T- and B-cell proliferation assay [59]. A natural *γ*-butyrolactone kinsenoside (Entry 31), originally isolated from *Anoectochillus roxburghii*, was reported as a potentially effective drug for treating patients with autoimmune hepatitis via targeting VEGFR2 to reduce the interaction between PI3K-AKT and JAK2-STAT pathways, which was confirmed in the vaccinated mouse model [60,61]. 

#### 2.2.6. Neuroprotective

Recent studies found that natural and synthetic *γ*-butyrolactones can be useful in the treatment of neurodegenerative disorders. Zhu and colleagues showed phenolic *γ*-butyrolactones in *Cinnamomum cassia* (Entry 32) exhibit a neuroprotective effect against tunicamycin-induced cell death in human dopaminergic neuroblastoma SH-SY5Y cells [62]. Guo and colleagues conducted similar studies and found that japonipene C (Entry 33) is responsible for the neuroprotective effect of the extract of *Petasites japonicas* [63]. Bi and colleagues revealed that the *γ*-butyrolactone derivative 3-benzyl-5-((2-nitrophenoxy)methyl)dihydrofuran-2(3*H*)-one (3BDO; Entry 34) protects against Aβ_25-35_-induced cytotoxicity in the PC12 cell. 3BDO was proposed to exhibit the protective effect by inhibiting ROS production and autophagy process [64]. In vivo assay was performed to evaluate memory rescuing activity as well as the Aβ lowering activity of 3BDO in mouse brain [65]. These findings show *γ*-butyrolactone can be utilized as potential therapeutic scaffold for the treatment of Parkinson’s disease and Alzheimer’s disease. 

#### 2.2.7. Antioxidant

The antioxidant activity of *γ*-butyrolactones has been verified using 1,1-diphenyl-2-picrylhydrazyl (DPPH) assay and superoxide scavenging assay. Lee and colleagues studied the antioxidant activity of styraxlignolide E (Entry 35) in *Styrax japonica* [66]. Boustie and colleagues found that norstictic acid (Entry 36) isolated from *Usnea articulate* shows superoxide scavenging activity higher than the well-known antioxidant quercetin [67]. The result suggested that this activity is involved in the antioxidant defense of lichens. 

#### 2.2.8. Hypoglycemic

The hyperglycemic activity of *γ*-butyrolactones has recently attracted attention as a possible therapeutic agent for type 2 diabetes. Lin and colleagues revealed that butyrolactone-1 (Entry 37) inhibits α-glucosidase in vitro and shows a potent TNF-α lowering effect [68]. The binding between butyrolactone-1 and α-glucosidase was theoretically proved in a molecular docking study. In an in vivo study on mice, potent hyperglycemic activity was maintained. Xiao and colleagues synthesized the analogues of butyrolactone-1 by modifying side chains (Entry 38) [69]. A biological evaluation showed that butyrolactone-1 derivatives display inhibitory activity of protein tyrosine phosphatase 1B (PTP1B) which is a promising therapeutic target of type 2 diabetes.

## 3. Synthesis of *γ*-Butyrolactones

### 3.1. Synthesis of γ-Butyrolactone via C5-O1 Bond Formation

#### 3.1.1. Oxidative Lactonization of Pentenoic Acid

The oxidative lactonization of alkenoic acid is one of the most popular transformations for the synthesis of lactone. A typical approach is usually initiated with the oxidation of olefin catalyzed by the highly toxic and expensive transition metal via the Prévost−Woodward reaction and Upjohn reaction conditions, and the subsequent intramolecular nucleophilic addition of carboxylic acid [70,71,72]. In contrast, recently reported methods for oxidative lactonization claimed metal-free and less toxic conditions, which utilized cheap and green organic catalysts and oxidants. These reactions have been developed with a view toward green chemistry.

**Figure 2:**
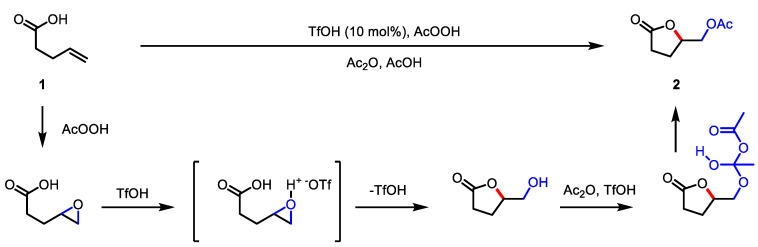
TfOH-catalyzed oxidative lactonization with peroxyacid.

In 2012, Gade and colleagues reported the triflic acid (TfOH)-catalyzed oxidative lactonization using peroxyacid as an oxidant (Figure 2) [73]. The cascade epoxidation of olefin **1** with peracetic acid and an intramolecular epoxide opening reaction provided *γ*-butyrolactone **2**. TfOH was proposed as a catalyst in both the ring-opening reaction via epoxide activation and acetylation of the subsequent hydroxyl group of *γ*-butyrolactone [74]. This method was applied to intramolecular lactonization as well as the intermolecular diacetylation of olefins. Considering the convenient process and the broad substrate scope, this might be an alternative approach to osmium tetroxide-catalyzed dihydroxylation of alkenes.

**Figure 3:**
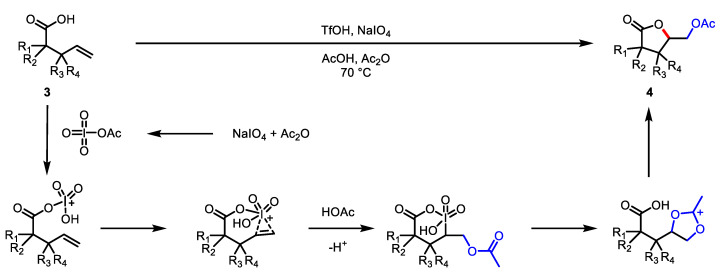
TfOH-catalyzed oxidative lactonization with sodium periodate.

Kang and colleagues also developed the TfOH-catalyzed oxidative lactonization of alkenoic acid **3** (Figure 3) [75]. Instead of peroxyacetic acid, sodium periodate was used as an oxidant. This method showed a high tolerance for a broad range of *α*,*β*-substituted pentenoic acid, providing the corresponding *γ*-butyrolactones **4** and bicyclic lactone scaffolds.

**Figure 4:**
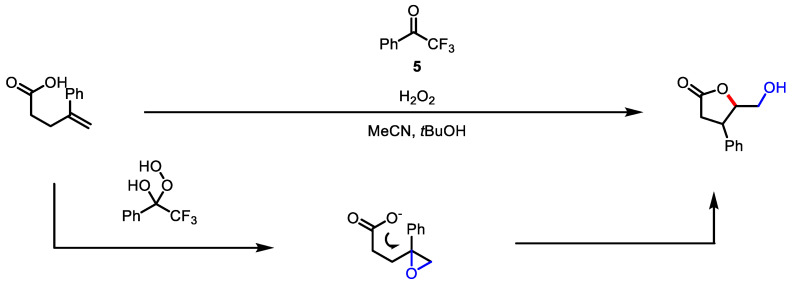
Trifluoroacetophenone-catalyzed oxidative lactonization with hydrogen peroxide.

Furthermore, Kokotos and colleagues developed an oxidative lactonization catalyzed by an organocatalyst, which relied on the use of hydrogen peroxide as the oxidant with 2,2,2-trifluoroacetophenone **5** as the organocatalyst (Figure 4) [76]. Mild reaction conditions led to an environmentally and industrially friendly process.

**Figure 5:**
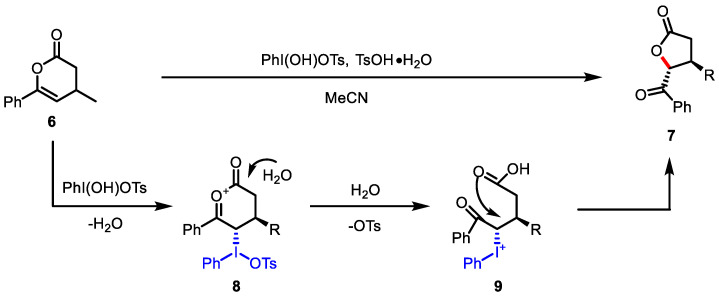
Oxidative ring contraction of 3,4-dihydropyran-2-ones.

The oxidative ring contraction strategy from 3,4-dihydropyran-2-ones **6** developed by Legault and colleagues using hypervalent iodine has been shown to provide 3,4-*trans*-*γ*-butyrolactones **7** (Figure 5) [77]. The authors suggested that the hyperiodine reagent selectively reacts with *trans*-face to *β*-substituents of **6**. This face selectivity generates iodinated intermediate **8** and the subsequent attack of a water molecule at the carbonyl position affords intermediate **9**. *γ*-butyrolactone **7** was diastereoselectively obtained through intramolecular substitution by carboxylic acid. The development of an enantioselective protocol was evaluated using a specific chiral iodine reagent.

**Figure 6:**
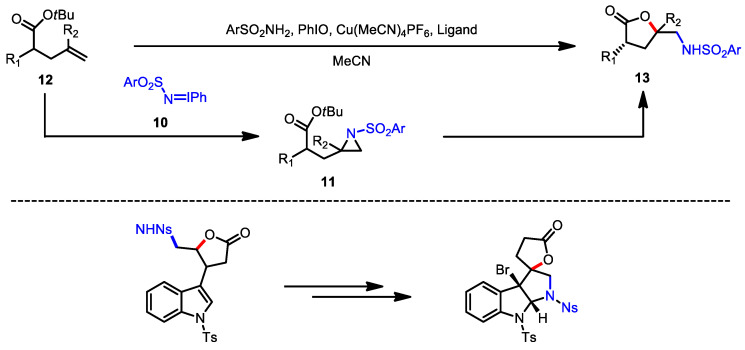
Aminolactonization of *t*-butyl pentenoate with iminoiodane (**top**) and the application of the resulting *γ*-butyrolactone (**bottom**).

As an analogous approach to oxidative lactonization, Dodd and colleagues reported aminolactonization with the use of in situ-generated nosyliminoiodane (Figure 6) [78]. The Cu-catalyzed generation of nitrene from arylsulfonyliminoiodane **10** was reported to yield aziridines from alkene groups [79,80]. For example, the aziridine intermediate **11**, generated after the metal-catalyzed reaction of *t*-butyl ester **12** with iminoiodane **10**, was successfully transformed into a high yield of amino *γ*-butyrolactone **13**. The usefulness of this aminolactonization was exemplified by further annulation of butyrolactone in novel complex heterocyclic systems (Figure 6, bottom).

#### 3.1.2. Halolactonization of Pentenoic Acid

The halolactonization of alkenyl carboxylic acids is widely used to construct functionalized lactone skeletons, including *γ*-butyrolactone. Generally, electrophilic NXS (e.g., NBS or NIS) and halogens are utilized to activate olefin moieties [81,82].

**Figure 7:**
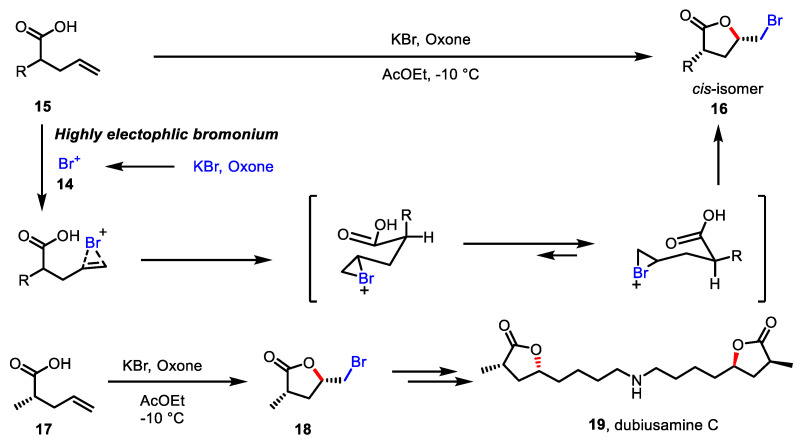
Bromolactonization of pentenoic acid with KBr and Oxone.

In 2011, Togo and colleagues developed a sustainable electrophilic bromine source via umpolung of alkali metal bromide [83]. Bromide (Br^-^) from potassium bromide, one of the most abundant and stable bromide sources, is oxidized into bromonium ion (Br^+^) **14** by oxidation with Oxone. Encouraged by the success of intramolecular bromo-amination with in situ-generated bromonium ion, the use of this umpolung system in the bromolactonization of 4-pentenoic acid **15** has been investigated, resulting in the production of *γ*-butyrolactone moieties **16** (Figure 7) [84]. At this stage, the preference of the diequatorial conformation of the transition state over the diaxial form results in the diastereoselective production of *cis*-isomer **16**. The utility of this approach was demonstrated by the total synthesis of dubiusamin C **19** from bromo butyrolactone **18**, which was obtained by the bromolactonization of pentenoic acid **17**.

**Figure 8:**
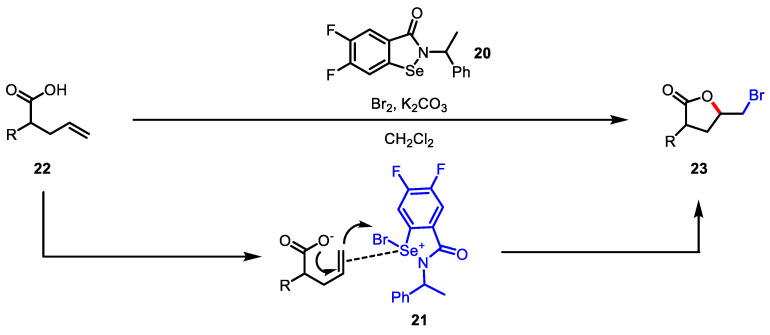
Bromolactonization of pentenoic acid with isoselenazolone.

Kumar and colleagues reported selenium-catalyzed bromolactonization by applying isoselenazolone **20** as a catalyst (Figure 8) [85]. Organoselenium compounds react with bromine to generate reactive bromoselenium intermediate **21**, which has a greater reactivity than NBS and molecular bromine (Br_2_) [86]. Several NMR studies confirmed that seleno-intermediate **21** plays a key role in the transfer of Br^+^ to the olefins of **22**. Intermediate **21** is catalytically regenerated in the presence of bromine or NBS with an inorganic base. This reaction allowed access to the construction of bromo butyrolactone **23** from a broad scope of pentenoic acids **22**.

#### 3.1.3. Acid-Promoted Cyclopropane Opening

The electrocyclic ring-opening reaction of cyclopropane has been demonstrated as a powerful tool for the construction of fused cyclic systems with sequential intramolecular trapping [87]. Several acid-catalyzed, domino cyclopropane opening/carboxylic acid trapping reactions have been investigated to construct fused-butyrolactone systems.

**Figure 9:**
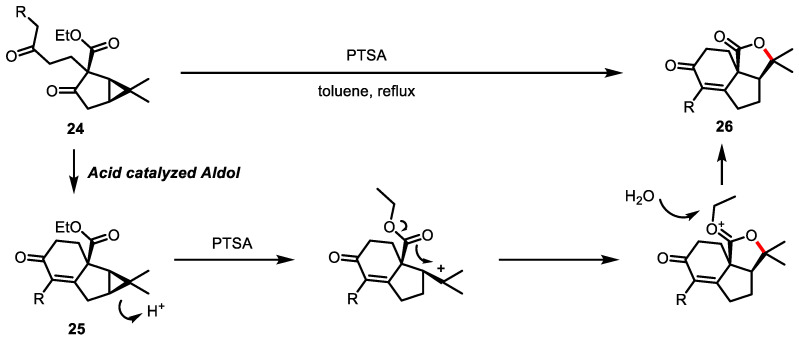
Acid-promoted cyclopropane opening/intramolecular ester trapping.

In 2017, Reddy and colleagues reported a Brønsted acid-catalyzed cascade reaction for the construction of a tricyclic structure **26** bearing a *γ*-butyrolactone core (Figure 9) [88]. This interesting reaction starts with *p*-toluenesulfonic acid (PTSA)-catalyzed aldol condensation of diketone **24** to afford bicyclic enone **25**, which subsequently undergoes acid-catalyzed cyclopropane opening/intramolecular trapping by an ester moiety.

**Figure 10:**
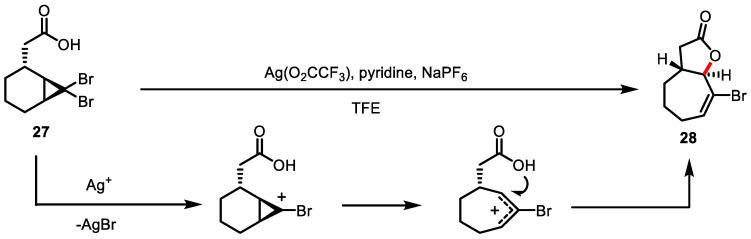
Silver-mediated cyclopropane opening/intramolecular acid trapping.

A similar domino reaction of silver (I)-mediated activation of dibromocyclopropane **27**/intramolecular acid trapping was developed by Batey and colleagues to form a *trans*-fused bicycle **28** possessing *γ*-butyrolactone (Figure 10) [89]. A unique *trans*-fused [5.3.0]-system presented in pseudoguainolide natural products was selectively obtained. Computational studies demonstrated the preference of a *trans*-fused system over a *cis*-fused system.

#### 3.1.4. Au-Catalyzed Oxaallylation

**Figure 11:**
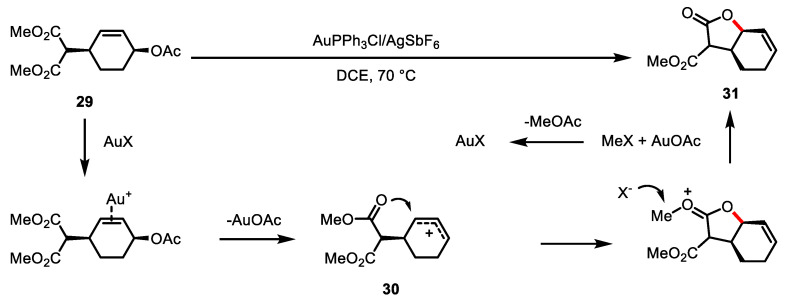
Gold-catalyzed intramolecular allylic alkylation of allylic acetate.

Gold-catalyzed allylic functionalization has been the object of diverse cyclization reactions and has been found to be efficient for the preparation of *γ*-butyrolactone [90,91,92]. Chen and colleagues examined the Au-catalyzed lactonization of allylic acetate **29** to construct a butyrolactone system (Figure 11) [93]. The proposed mechanism involved the generation of an allylic cation intermediate **30** from allylic acetate **29** in the presence of the Au catalyst. The subsequent nucleophilic attack by the ester moiety resulted in the formation of bicyclic *γ*-butyrolactone **31**.

**Figure 12:**
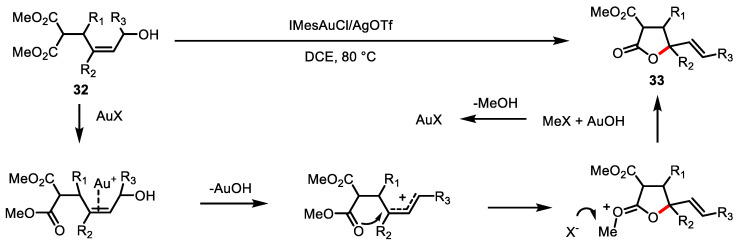
Gold-NHC complex catalyzed intramolecular allylic alkylation of allylic alcohol.

Bandini and colleagues reported the direct activation of free allylic alcohol **32** by applying a gold catalyst with *N*-heterocyclic carbene (Figure 12) [94]. An allylic cation intermediate is generated upon coordination of the NHC-gold complexes to a free allylic alcohol **32**. The resulting poly-substituted *γ*-butyrolactone **33** was obtained via nucleophilic attack by ester and subsequent dealkylation.

**Figure 13:**
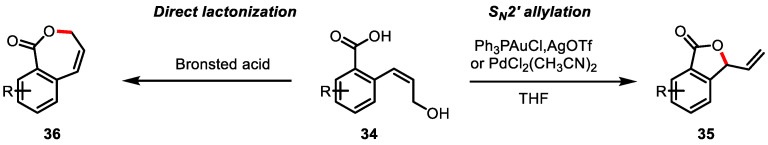
Gold-catalyzed dehydrative lactonization.

More recently, Aponick and colleagues developed a gold-catalyzed oxa-allylation of a free allyl alcohol **34** with an intramolecular free carboxylic acid to prepare *γ*-butyrolactone **35** (Figure 13) [95]. In contrast to Brønsted acids generating a 7-membered lactone skeleton **36** via direct acid-catalyzed esterification, *γ*-butyrolactone **35** was obtained using a transition-metal catalyst via an S_N_2′-type oxa-allylation mechanism.

**Figure 14:**
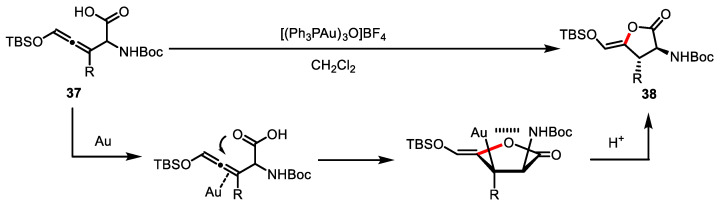
Gold-catalyzed lactonization of allene system.

Allenylglycine **37** was also used as a precursor for the construction of *γ*-butyrolactone **38**. Ohfune and colleagues applied the Au-catalyzed intramolecular lactonization into the allene system **37**, which is a useful substrate for gold catalysis (Figure 14) [96]. Interestingly, *γ*-butyrolactone **38** was obtained regio- and diastereoselectively via 5-endo-dig cyclization in the presence of bulky TBS at the allenic terminal carbon.

#### 3.1.5. Photoredox-Catalyzed Lactonization

Photoredox catalysis through single-electron transfer (SET) has attracted significant attention in the community of organic chemistry. Not surprisingly, the application of photoredox catalysis to the ring formation reaction, including *γ*-butyrolactone synthesis, has been intensively explored. As shown in Table 3, several synthetic approaches have been reported to provide 5,5-disubstituted *γ*-butyrolactone.

**Figure 15:**
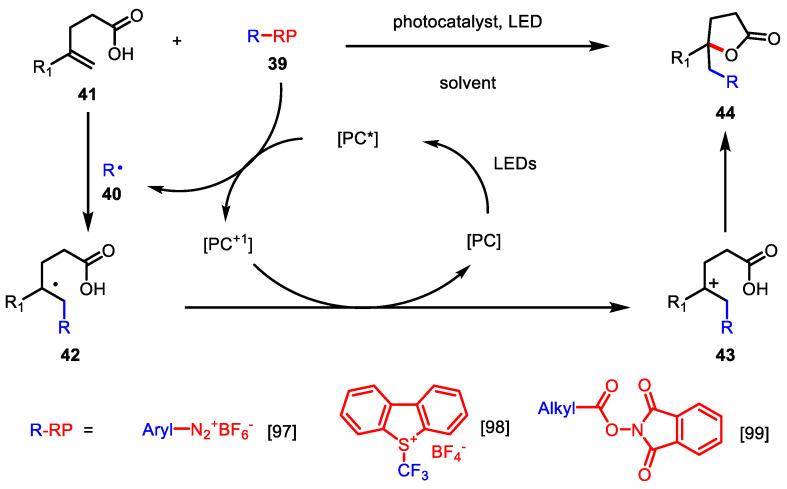
Photoredox-catalyzed γ-butyrolactone synthesis.

Photoredox-catalyzed *γ*-butyrolactone synthesis generally starts with radical generation through the reduction of radical precursors **39** (e.g., diazonium salt, *N*-hydroxylphthalimide ester, etc.) by the oxidative quenching of the excited state of the photocatalyst (PC *). The in situ-generated radical **40** adds to the alkene of **41** to produce intermediate **42**, which is transformed to carbocation **43** through single-electron transfer (SET) with an oxidized photocatalyst (PC^+^). Nucleophilic attack of the carboxylic acid results in the *γ*-butyrolactone **44** (Figure 15). Aryl diazonium salts (Entry 1) [97], Umemoto’s reagent (Entry 2) [98], *N*-hydroxylphthalimide ester (Entry 3) [99], and α-bromo ester [100] were used in these reactions.

### 3.2. Synthesis of γ-Butyrolactone via C4-C5 and C2-O1 Bonds Formation

Connecting the C4-C5 bond in [3 + 2] annulation-type *γ*-butyrolactone formation is one of the most promising routes. Retrosynthetically, the disconnection of the C4-C5 and C2-O1 bonds gives a^3^ and a^2^ synthons; thus, this mismatched relationship should be overcome through a certain umpolung reaction.

#### 3.2.1. Transition-Metal Catalyzed C-C Bond Coupling

**Figure 16:**
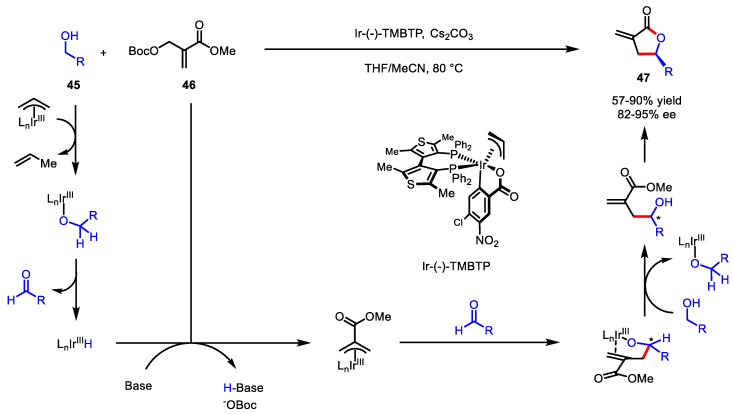
Asymmetric synthesis of *α*-exo-methylene-*γ*-butyrolactone via iridium-catalyzed 2-(alkoxycarbonyl)allylation.

Krische et al. applied their transfer hydrogenative C-C bond coupling chemistry to the *γ*-butyrolactone syntheses. In 2012, they reported that the iridium-catalyzed carbonyl 2-(alkoxycarbonyl)allylation between various primary alcohols **45** and acrylic ester **46** afforded *γ*-substituted *α*-exo-methylene-*γ*-butyrolactone **47** with high enantioselectivity (Figure 16) [101]. As shown in the mechanism, this transformation involves an a^3^–d^3^ umpolung process regarding the *β*-position of the acrylate counterpart **46**, which normally acts as an electrophile during C-C bond-forming reactions [102].

**Figure 17:**
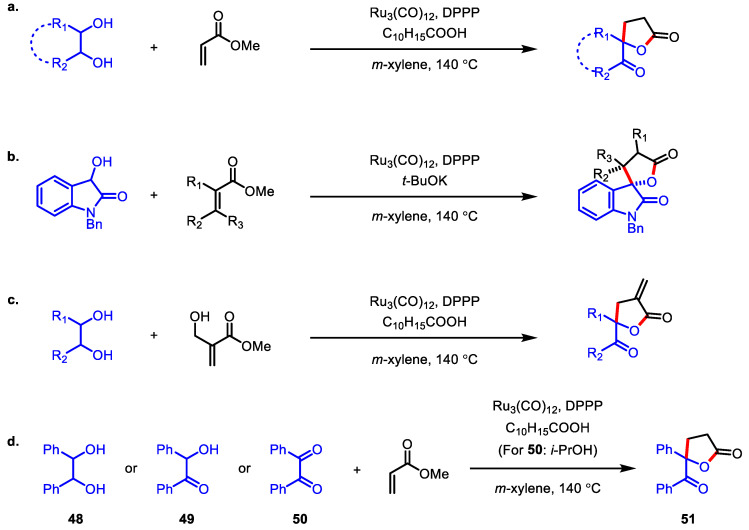
Syntheses of *γ*-butyrolactones via ruthenium-catalyzed hydrohydroxyalkylation. (**a**) Syntheses of spiro-*γ*-butyrolactones from diols and methyl acrylate; (**b**) Syntheses of polysubstituted 2,3’-spirooxindole-*γ*-butyrolactones from *N*-benzyl-3-hydroxyoxindole and acrylic esters; (**c**) Syntheses of *α*-exo-methylene-*γ*-butyrolactones from hydroxyl-substituted methacrylate and diols; (**d**) Redox level-independent formation of **51**.

In the C-C bond constructing catalytic transfer hydrogenation, a secondary alcohol was not a suitable partner of acrylates because of the low susceptibility to the nucleophilic attack [103] of the π-allyl complex derived from the acrylates. Just a year after their first report, Krische and colleagues also revealed that ruthenium(0)-catalyzed hydrohydroxyalkylation of acrylates with vicinal diols or their oxidized congeners could provide a series of *γ*-butyrolactones, including spiro-*γ*-butyrolactones (Figure 17a), polysubstituted 2,3′-spirooxindole-*γ*-butyrolactones (Figure 17b), and *α*-exo-methylene-*γ*-butyrolactones (Figure 17c) [104]. As illustrated in Figure 17d, 1,2-diol **48** and its highly oxidized congeners **49** and **50** were transformed into the same outcome **51**, indicating that this transformation proceeds in a redox level-independent manner.

**Figure 18:**
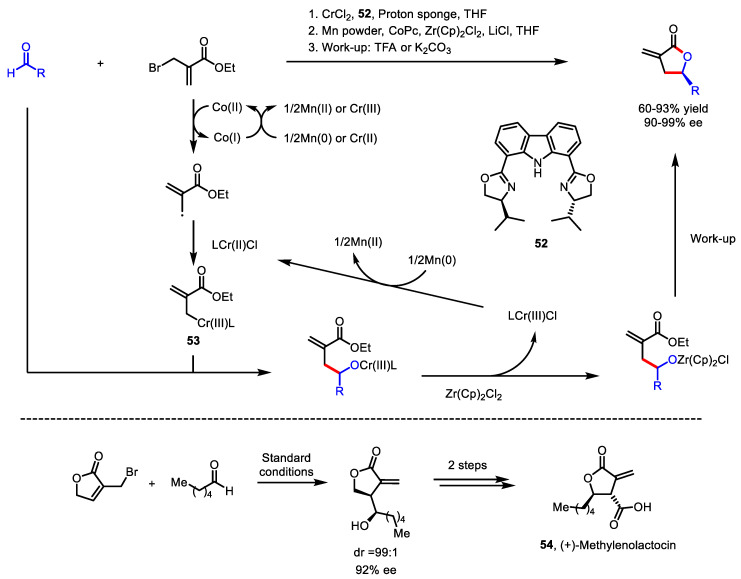
Asymmetric synthesis of *α*-exo-methylene *γ*-butyrolactone via chromium-catalyzed 2-(alkoxycarbonyl)allylation and lactonization and total synthesis of (+)-methylenolactocin.

The asymmetric synthesis of *α*-exo-methylene *γ*-butyrolactones was developed by Zhang and colleagues in 2015 (Figure 18) [105]. This methodology utilized an enantioselective chromium-catalyzed carbonyl 2-(alkoxycarbonyl)allylation of a wide range of aldehydes. To achieve superior enantioselectivity, the C2 symmetric bisoxazoline ligand was essential. Rigidification of Guiry’s tridentate ligand [106] provided a new ligand **52**, which resulted in excellent enantiomeric excess of up to 99%. Similar to the previous methods [101,104], the inherent positive character of the acrylate *β*-position was inverted via the cobalt-assisted generation of allyl-chromium species **53**. To demonstrate the synthetic utility, the total synthesis of an antitumor and antimicrobial natural product, (+)-methylenolactocin **54,** was successfully conducted with a 53% overall yield over three steps and 92% ee. (Figure 18, bottom).

**Figure 19:**
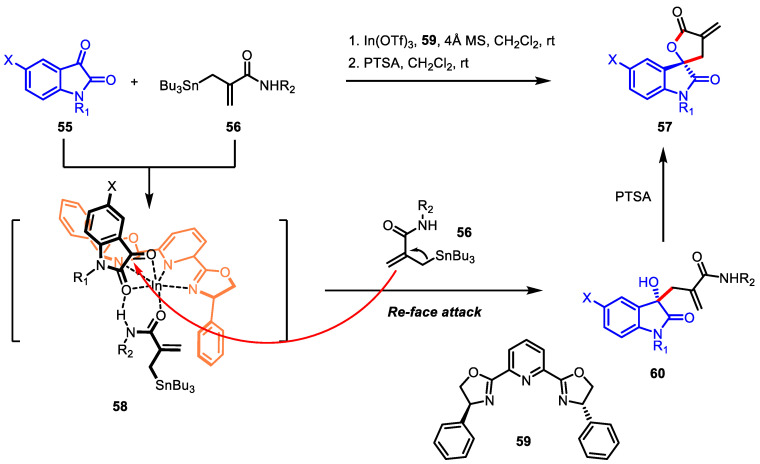
Asymmetric synthesis of 2,3′-spirooxindole-*α*-exo-methylene *γ*-butyrolactone via indium-catalyzed amide allylation and lactonization.

Spirooxindoles [107] and *α*-exo-methylene-*γ*-butyrolactones [12,108], biologically relevant structural motifs, have received attention from medicinal chemists. In this regard, the fusion of two scaffolds would be a promising strategy for securing biologically active scaffolds. In 2013, the first asymmetric synthesis of 2,3′-spirooxindole-*α*-exo-methylene *γ*-butyrolactone **57** via the indium(III)-catalyzed allylation of isatins **55** and *β*-amido allylstannanes **56** was reported (Figure 19) [107,109]. The amide NH proton of allylstannanes was essential for enhancing enantioselectivity as well as complete conversion by engaging in six-coordinated indium complex **58** with tridentate ligand **59**, thereby inducing **56** to approach from *Re*-face [109]. The resulting acyclic 2-oxindoles **60** was cyclized under acidic conditions to afford the desired lactone **57** with complete stereochemistry retention.

#### 3.2.2. NHC-Catalyzed C-C Bond Coupling

A chiral *N*-heterocyclic carbene (NHC) has played an important role in making a homoenolate nucleophile from enals through the a^3^–d^3^ umpolung reaction [110]; thus, it has been widely used in the optically active *γ*-butyrolactone synthesis via [3 + 2] annulation. Over the last decade, this strategy has been employed to construct a 2,3′ spirooxindole-*γ*-butyrolactone system.

**Figure 20:**
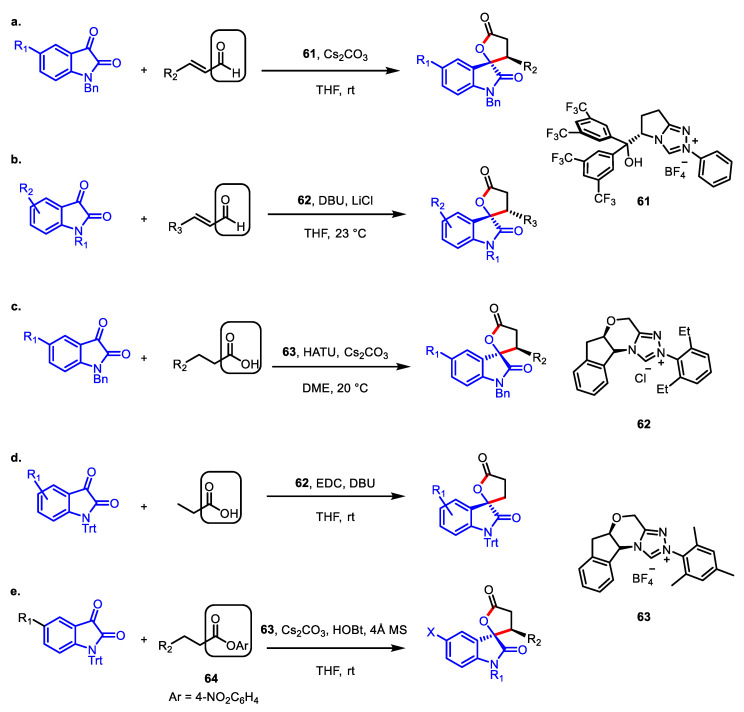
Asymmetric syntheses of 2,3′-spirooxindole-*γ*-butyrolactone via NHC-catalyzed homoenolate annulation. (**a**,**b**) NHC-catalyzed 2,3′-spirooxindole-*γ*-butyrolactone formation from enals; (**c**,**d**) NHC-catalyzed 2,3′-spirooxindole-*γ*-butyrolactone formation from carboxylic acids; (**e**) NHC-catalyzed 2,3′-spirooxindole-*γ*-butyrolactone formation from aryl esters.

In 2011, Ye and colleagues discovered the first enantioselective NHC-catalyzed synthesis of spirooxindole-*γ*-lactone with isatin and an enal as substrates (Figure 20a) [111]. A chiral NHC **61** derived from l-pyroglutamic acid displayed the best result, affording the desired spirolactone up to 99% ee. A proximal hydroxy group in **61** was crucial to obtain the lactone with an excellent yield and enantioselectivity because the hydrogen bonding between the carbonyl group of isatin and the catalyst hydroxy group may guide the direction of the isatin approach and enhance its reactivity.

A year later, a similar NHC-catalyzed transformation was carried out in the presence of lithium chloride as an external activator. Scheidt and colleagues revealed that the addition of two equivalents of LiCl to the reaction gave the beneficial effect of creating an organized transition state with **62**, which offered excellent enantioselectivity, similar to the role of the internal hydroxy group of **61** in the previous method (Figure 20b) [112].

In 2015, it was independently disclosed by Chi (Figure 20c) [113] and Yao (Figure 20d) [114] that aliphatic acids could participate in the NHC-catalyzed spiro-*γ*-lactone construction instead of the aldehyde substrates. The key to this modification was the in situ pre-activation of carboxylic acid by various peptide coupling reagents, which enabled the formation of a common NHC-coupled homoenolate intermediate.

Finally, Xu and colleagues reported that the saturated aryl ester **64** was also able to engage in this type of NHC-catalyzed asymmetric annulation with catalytic amount of 1-hydroxybenzotriazole (HOBt) (Figure 20e) [115]. After the experimental studies, it was revealed that HOBt had a dual role: activation of the ester for the next substitution by the chiral NHC, and the stabilization of the effective transition state via hydrogen bonding.

**Figure 21:**
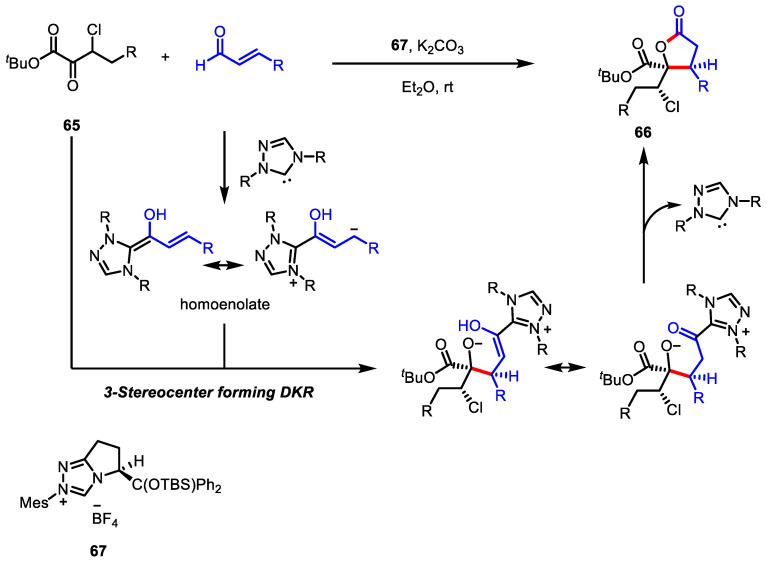
Asymmetric synthesis of 3,4,4-trisubstituted *γ*-butyrolactones via NHC-catalyzed dynamic kinetic resolution.

A chiral NHC led to significant advances in dynamic kinetic resolution (DKR)-mediated asymmetric transformation. In 2015, Johnson and colleagues developed the first intermolecular DKR between *α*,*β*-unsaturated aldehydes and racemic *β*-halo-*α*-keto esters **65**, which installed three stereocenters during the single bond-forming process (Figure 21) [116]. Using this strategy, they obtained 3,4,4-trisubstituted *γ*-butyrolactones **66** with three contiguous stereocenters in a single operation, with excellent enantioselectivity (up to 98% ee).

#### 3.2.3. Photoredox-Catalyzed C-C Bond Coupling

**Figure 22:**
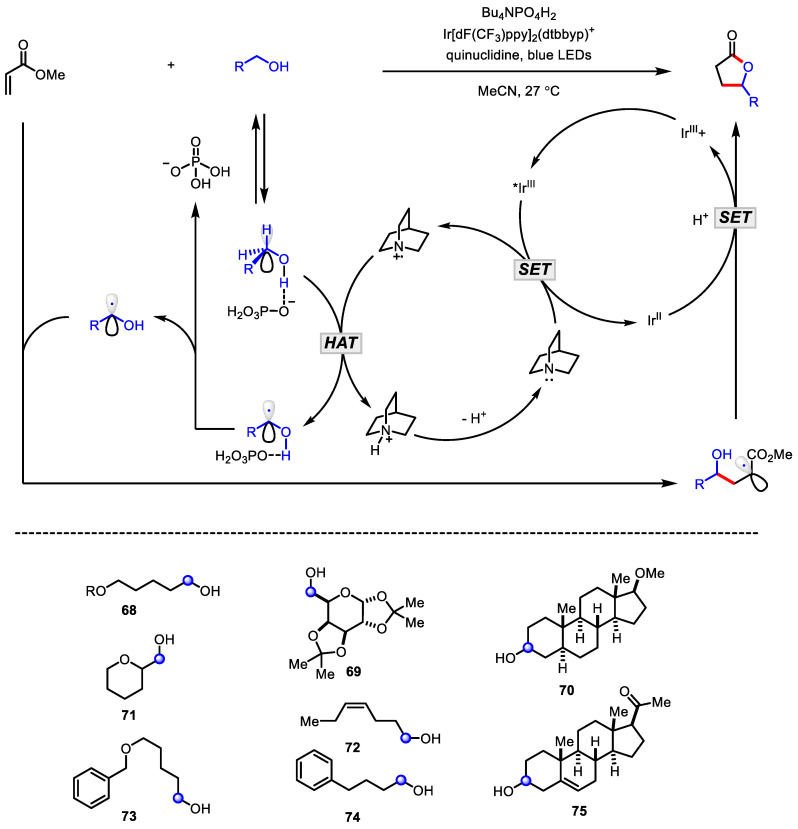
Synthesis of *γ*-butyrolactones via the alcohol-selective C-H activation mediated by photoredox catalysis.

Photoredox catalysis achieves the cutting-edge evolution in the C-H bond activation chemistry; thus, it enables not only mild, economical, and environmentally friendly chemical reactions, but also the discovery of unprecedented reactivity of chemical bonds [117]. In 2015, MacMillan’s seminal work demonstrated that the *α*-C–H bond of alcohols could by selectively activated in the presence of allylic, benzylic, *α*-C=O, and *α*-ether C-H bonds. In addition, the corresponding *α*-hydroxyl radical participated in the formation of the *γ*-lactones with methyl acrylate (Figure 22) [118]. The C–H bond-weakening, assisted by hydrogen bond, gave rise to the unique selectivity, which was supported by tetra-*n*-butylammonium phosphate as a catalytic H-bond acceptor. The versatility of this methodology was demonstrated by testing several structurally complex substrates **68**–**75** containing inherently activated C–H bonds (Figure 22, bottom).

**Figure 23:**
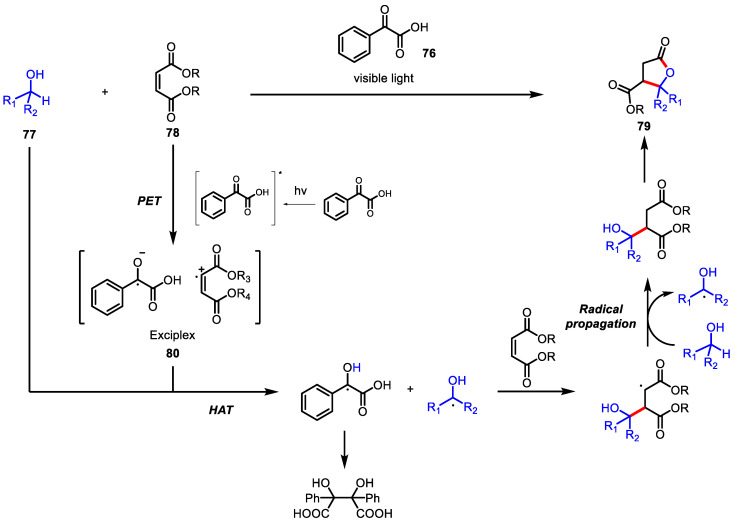
Synthesis of *γ*-butyrolactones via photoorganocatalytic C-H activation.

Recently, the greener variant of typical photoredox catalysis, the photo-organocatalytic synthesis of this lactone has been accomplished by Kokotos and colleagues. (Figure 23) [119]. By utilizing a readily available and cheap photoinitiator, phenylglyoxalic acid **76** as an alternative to transition metal catalysts, a variety of primary and secondary alcohol **77** and a maleic acid diester **78** merged into the corresponding *γ*-butyrolactones **79** in the presence of visible light from sunlight or simple household lamps. Through extensive mechanistic studies, it was proposed that photoinduced exciplex **80** formation facilitates selective hydrogen atom abstraction from the secondary alcohol.

#### 3.2.4. Miscellsious γ-Butyrolactone Formation

**Figure 24:**
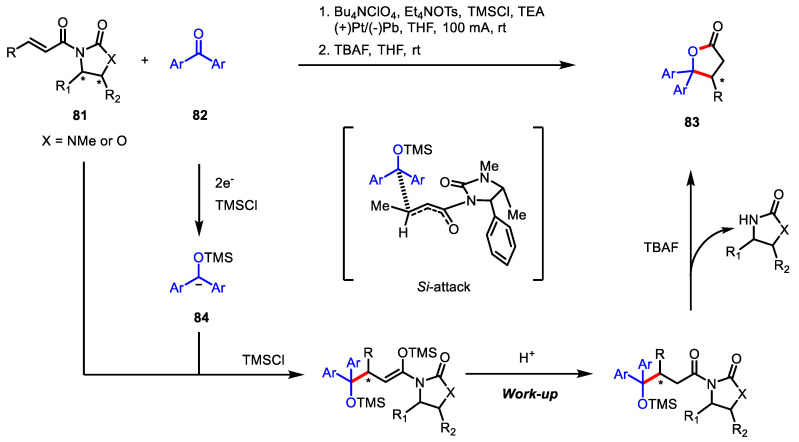
Asymmetric synthesis of 4,5,5-trisubstituted-*γ*-butyrolactones via electroreductive C-C bond coupling.

Electroreduction of carbonyl compounds can convert electrophilic carbonyl compounds into nucleophilic carbanion, which is further involved in the [3 + 2] coupling of *γ*-butyrolactones. In this regard, electroreductive C-C coupling of *α*,*β*-unsaturated carbonyl compounds with ketones or aldehydes has been known to be useful for the synthesis of *γ*-butyrolactones. A previous electroreductive method [120] toward lactones in the presence of trimethylsilyl chloride (TMSCl) was improved by Kise and colleagues by means of a chiral auxiliary, leading to optically active 4,5,5-trisubstituted *γ*-butyrolactones **83** in high diastereoselectivity (Figure 24) [121]. The reaction is initiated with two-electron transfer to a more reducible diaryl ketone **82.** The resulting carbanion **84** is diastereoselectively coupled with the Michael acceptor **81**. DFT calculations for the bond-forming transition states explained the reason for its *Si*-face preference.

**Figure 25:**
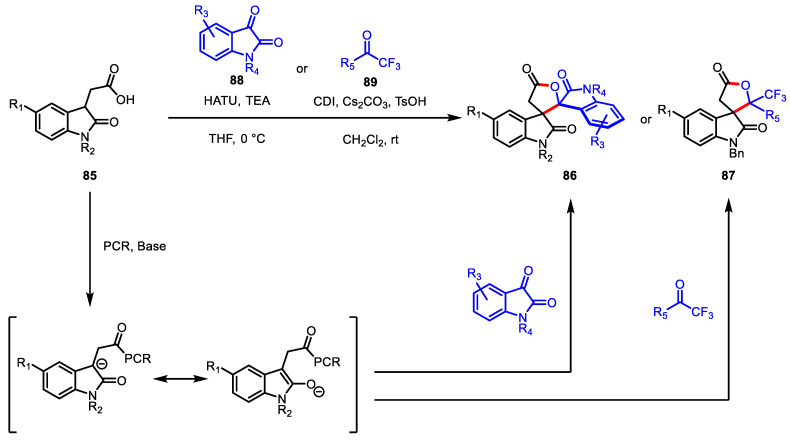
Synthesis of 3,3′-spirooxindole-*γ*-butyrolactones via peptide coupling reagent-assisted lactonization.

The synthesis of 3,3′-spirooxindole-*γ*-butyrolactones, another isomeric form of the spirooxindole-*γ*-lactone motif, has attracted less attention, but it is still valuable when it comes to the longstanding need to secure a structurally diverse chemical library in the drug discovery field. In 2017, Du and colleagues revealed that the peptide coupling reagent (PCR)-assisted *β*-functionalization of indoline-2-one aliphatic acids **85** could produce the desired spirofused *γ*-lactone **86** and **87** via [3 + 2] coupling with electrophilic carbonyl substrates; isatins **88** or trifluoromethyl ketones **89** (Figure 25) [122]. After the intensive screening of the reaction conditions, it was found that the optimal PCR was HATU for isatin substrates and CDI for trifluoromethyl ketone substrates.

**Figure 26:**
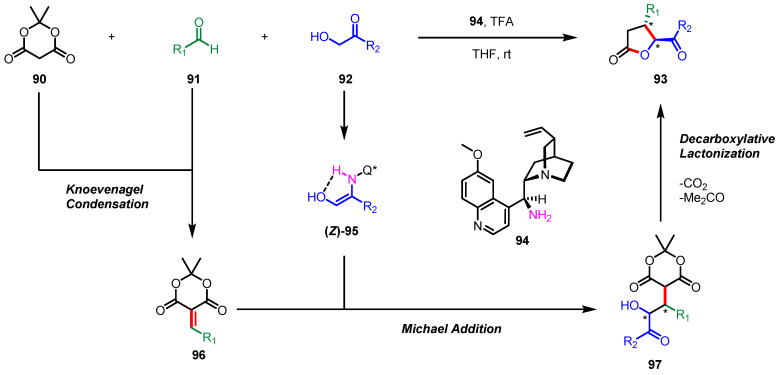
Asymmetric synthesis of 4,5-disubstituted-*γ*-butyrolactones via organocatalyzed three-component coupling.

In 2017, a one-pot multicomponent reaction was exploited to construct enantiomerically pure 4,5-disubstituted *γ*-butyrolactones **93** by Bhat and colleagues. (Figure 26) [123]. Their strategy was the organocatalyzed Knoevenagel condensation/Michael addition/decarboxylative lactonization cascade utilizing cheap and readily accessible starting materials such as Meldrum’s acid **90**, aldehydes **91**, hydroxyketones **92**, and the chiral cinchona catalyst **94**. Enamine (*Z*)-**95**, which has a chiral environment induced by **94**, is subjected to asymmetric 1,4-addition with the Knoevenagel condensation adduct **96** to afford **97** bearing two contiguous stereogenic centers. This precisely designed three-component reaction was able to avoid possible side reactions such as aldol condensation products between **91** and **92**.

### 3.3. Synthesis of γ-Butyrolactones via C3-C4 and C2-O1 Bond Formation

Connecting the C3-C4 bond in [3 + 2] annulation-type *γ*-butyrolactone formation has been less investigated than that of C4-C5 bond formation. Nevertheless, the development of this synthetic route is still significant, in that securing diverse synthetic tools has always been beneficial to organic chemists, particularly for complex natural product synthesis. Retrosynthetically, the disconnection of the C3-C4 and C2-O1 bonds gives d^3^ and d^2^ synthons; thus, this mismatched relationship should be overcome through a certain umpolung reaction.

**Figure 27:**
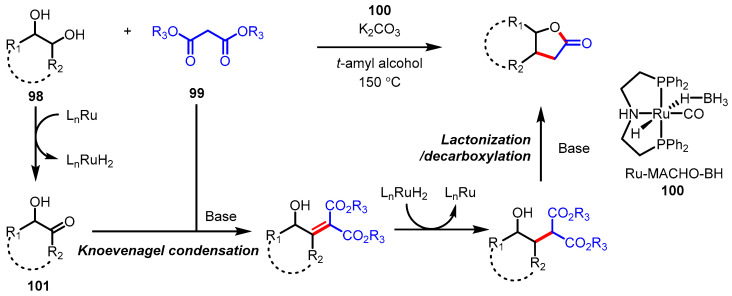
Synthesis of *γ*-butyrolactones via ruthenium pincer-catalyzed hydrogen autotransfer.

A borrowing hydrogen methodology, also known as hydrogen autotransfer, is a subclass of a wide range of transfer hydrogenation chemistry similar to the aforementioned transfer hydrogenative C–C bond coupling [124,125]. Beller and colleagues reported that ruthenium (Ru) pincer catalyst **100** promoted *γ*-butyrolactone synthesis from 1,2-diols **98** and malonates **99** (Figure 27) [126]. Catalyst **100** temporarily abstracts hydrogen from 1,2-diols to give the corresponding *α*-hydroxyketone **101**, which can act as an electrophile. This step belongs to a polarity inversion process at the C3 position of the resulting *γ*-lactones. Whereas the above-mentioned Ru-catalyzed spirolactonization consequentially delivers alcohol C–H functionalization type products (see Figure 17), this Ru-catalysis proceeds through a type of alcohol substitution, which offers monocyclic lactones.

**Figure 28:**
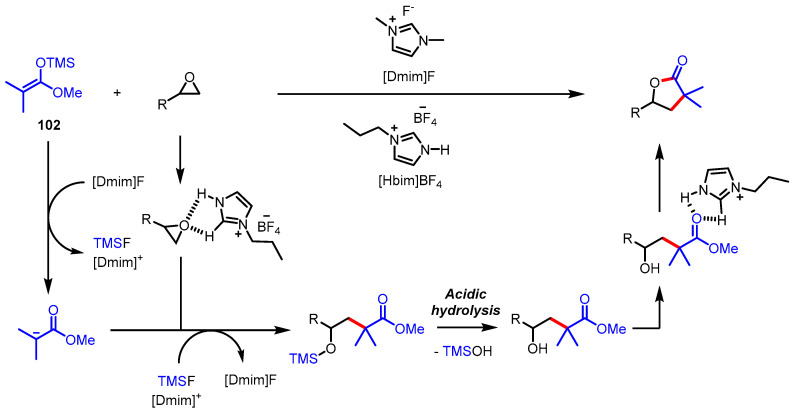
Synthesis of *γ*-butyrolactones via ionic liquid-assisted epoxide opening and lactonization.

An epoxide is a useful three-atom building block in the [3 + 2] annulation strategy because of its susceptibility to the attack of suitable carbon nucleophiles such as ester enolates. In 2017, ketene silyl acetal **102** was applied as the effective enolate equivalent to constructing the lactone via regioselective epoxide opening followed by lactonization (Figure 28) [127]. Additionally, an ionic liquid system composed of a mixture of 1,3-dimethylimidazolium fluoride ([Dmim]F) and 1-butylimidazolium tetrafluoroborate ([Hbim]BF_4_) was utilized to achieve the desired transformation. The catalytic amount of [Dmim]F acted as a Si-O bond activator and [Hbim]BF_4_ served as the solvent providing acidic media. This ionic liquid mixture was able to be reused up to three times, which is valuable for the contribution toward green chemistry.

### 3.4. Synthesis of Butyrolactone via C3-C4 and C5-O1 Bonds Formation

There are a few examples of this synthetic approach through the formation of C3-C4 and C5-O1 bonds during 2010 to 2020. Mostly, the single-electron transfer pathway is involved in the C3-C4 and C5-O1 bond formation approaches. First, photoredox catalysis was applied with alkenes and suitable counterparts such as *α*,*β*-unsaturated acid [128], oxime acid [129], or haloacetic acid [130]. Second, a metal oxidant-mediated transformation of glycals to *γ*-butyrolactones was reported [131]. Third, the copper-catalyzed-cyclopropanol ring-opening cross-coupling reaction was utilized to synthesize *γ*-butyrolactones containing quaternary carbon centers [132].

#### 3.4.1. Polar Radical Crossover Cycloaddition (PRCC)

**Figure 29:**
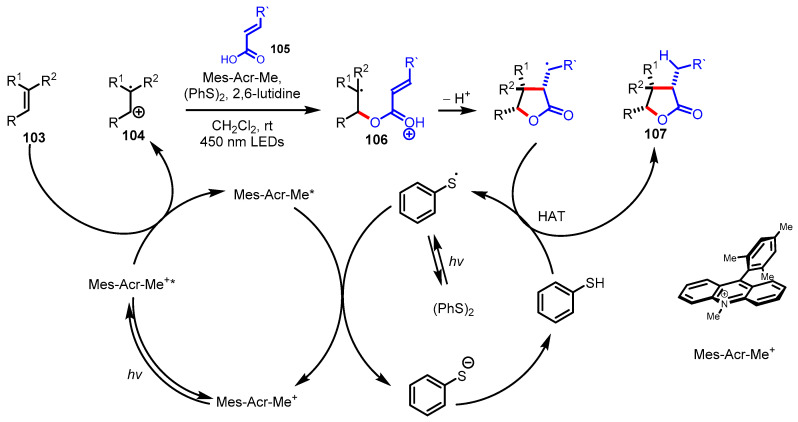
Polar radical crossover cycloaddition of the oxidizable alkenes and *α,β*-unsaturated acids.

**Figure 30:**
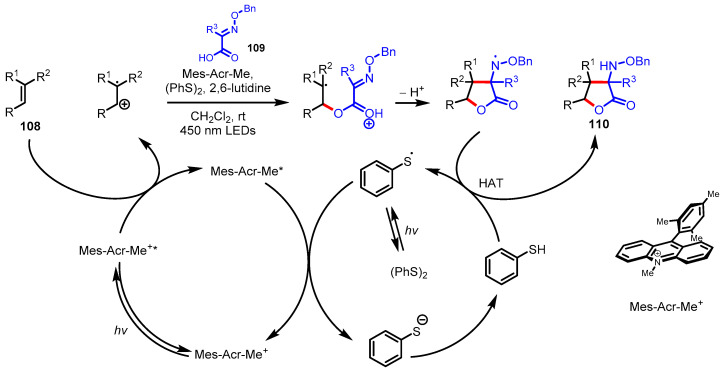
Polar radical crossover cycloaddition of the oxidizable alkenes and *O*-benzyloxime acids.

Polar radical crossover cycloaddition (PRCC) has been utilized in the construction of various saturated heterocycles, including tetrahydrofurans [133], *γ*-lactams, and pyrrolidines [134]. The co-catalyst of Fukuzumi acridinium single-electron photooxidant and a redox-active hydrogen atom donor is a key mediator of PRCC through photoredox catalysis. Nicewicz and colleagues extended the PRCC approach to the synthesis of *γ*-butyrolactones [128]. First, the oxidizable alkenes **103** and *α*,*β*-unsaturated acids **105** as nucleophiles forged *γ*-butyrolactones **107** under photoredox catalysis. As depicted in Figure 29, an electrophilic alkene cation radical **104** is formed by the excited acridinium-mediated single-electron oxidation followed by the generation of the radical intermediate **106** through the addition of carboxylic acid **105** to the alkene cation radical. 5-exo-trig radical cyclization and hydrogen atom transfer with thiophenol provided the desired *γ*-butyrolactones. Alternatively, α-amino-*γ*-butyrolactones **110** have also been synthesized by the PRCC method using oxidizable alkenes **108** and *O*-benzyloxime acids **109**, which correspond to *α*,*β*-unsaturated acids **105** (Figure 30) [129].

#### 3.4.2. Atom-Transfer Radical Addition (ATRA)

**Figure 31:**
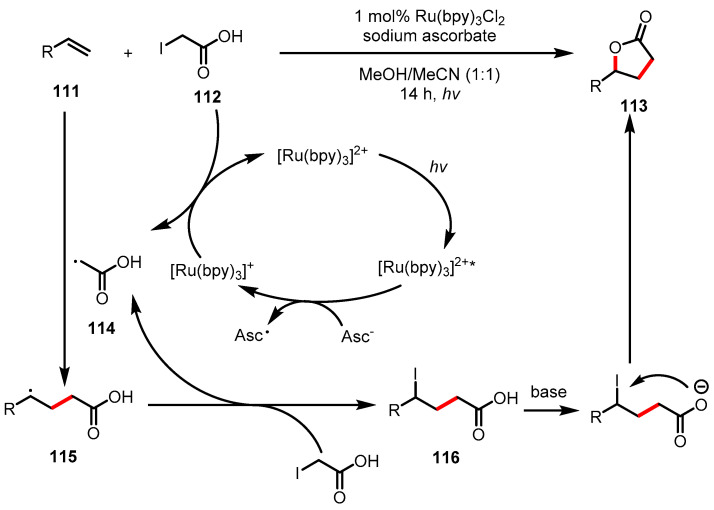
*γ*-Butyrolactone synthesis via the photoredox-catalyzed atom-transfer radical addition (ATRA).

Another example of *γ*-butyrolactone synthesis mediated by photoredox catalysis is atom-transfer radical addition (ATRA), which was reported by Kokotos and colleagues in 2018 [130]. ATRA has been utilized as a powerful method for one-step C-C and C-X bond formation between olefins and haloalkanes. Kokotos and colleagues applied photoredox catalysis in ATRA using Ru(bpy)_3_Cl_2_ as a photoredox catalyst, which was employed in the conversion of alkenes **111** and α-iodoacetic acids **112** to *γ*-butyrolactones **113** under light irradiation. In this reaction, the excited photocatalyst is reduced by ascorbate, followed by reaction with *α*-iodoacetic acid **112** to generate the electrophilic radical **114**, which reacts with the alkene leading to radical **115**. Then, propagation proceeded with iodoacetic acid, resulting in the formation of **116**. Finally, *γ*-butyrolactone **113** is formed by the deprotonated carboxylic acid under basic reaction conditions (Figure 31).

#### 3.4.3. Mn(OAc)_3_-Mediated Radical Lactonization

**Figure 32:**
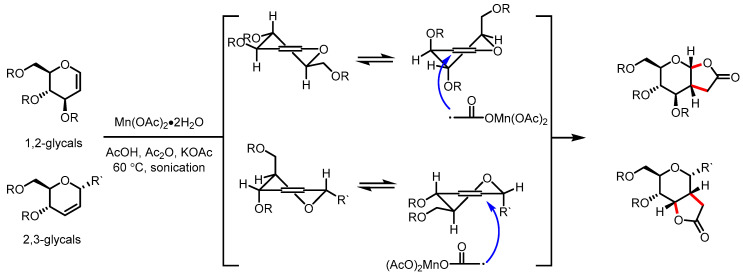
Synthesis of carbohydrate-based *γ*-butyrolactones through Mn(OAc)_3_-mediated radical lactonization.

Manganese (III) acetate has been utilized as a versatile single-electron transfer (SET) reagent. Mukherjee and colleagues reported Mn(OAc)_3_-mediated radical lactonization to synthesize carbohydrate-based *γ*-butyrolactones from glycals [131]. Under sonication, a variety of 1,2-glycals and 2,3-glycals were converted to *γ*-butyrolactones in a regioselective and stereoselective manner, which were governed by conformational preferences for glycal substrates (Figure 32).

#### 3.4.4. Copper-Catalyzed Cyclopropanol Ring-Opening Cross-Coupling Reaction

**Figure 33:**
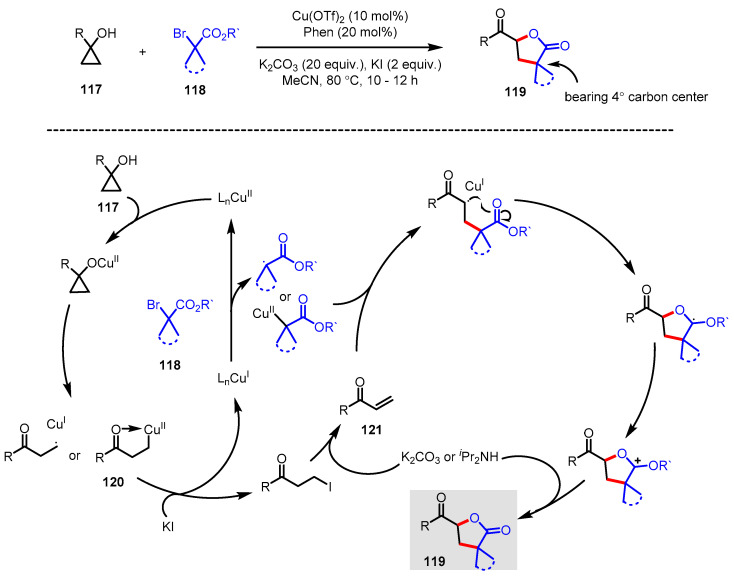
Synthesis of *γ*-butyrolactones bearing quaternary carbon centers via copper-catalyzed cyclopropanol ring-opening cross-coupling reaction.

Cyclopropanols **117** are versatile substrates in various ring-opening and ring-expansion reactions because of the intrinsic stain of the three-membered ring. One of the representative reactions in this class is the cyclopropanol ring-opening cross-coupling reaction mediated by diverse transition metal catalysts or single-electron transferring oxidants, resulting in the formation of a variety of *β*-substituted ketones. Formation of *α*,*β*-unsaturated enone byproducts, which are normally caused by *β*-hydride elimination of the metallo-homoenolate **120**, is one of the major issues in this reaction. Interestingly, Dai and colleagues developed a method to accelerate *α*,*β*-unsaturated carbonyl byproduct **121** by adding potassium iodide in the reaction mixture and reacting with 2-bromo-2,2-dialkyl acetate **118** to obtain *γ*-butyrolactones **119** bearing quaternary carbon centers, which are catalyzed by Cu(OTf)_2_ (Figure 33) [132].

### 3.5. Synthesis of γ-Butyrolactones via C2-C3 and C2-O1 Bonds Formation

Carbon monoxide is used as a versatile C1 source in organic synthesis, thereby reacting with suitable unsaturated alcohols to afford various ring sizes of lactones [135]. There have been increasing reports of methodologies for producing *γ*-butyrolactones using carbonylations and hydroformylations over the past decades. However, due to the innate drawbacks of CO, including its high toxicity, gaseous nature, and strict regulations for transportation, bypassing the direct use of CO gas is another significant topic in carbonylation research [135].

#### 3.5.1. Carbonylative Lactonization

**Figure 34:**
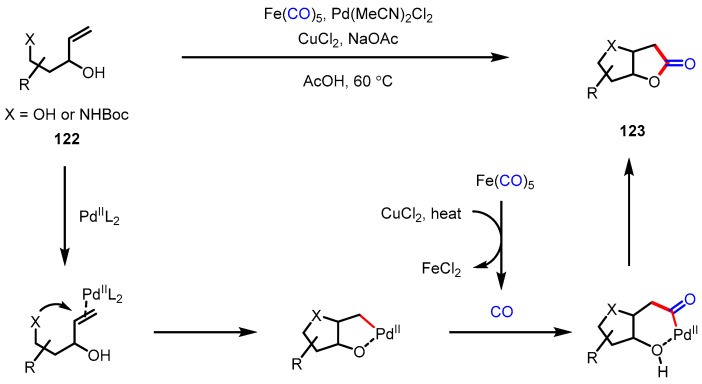
Synthesis of bicyclic *γ*-butyrolactones via palladium-catalyzed carbonylation using iron pentacarbonyl.

Among various methodologies utilizing CO gas or other carbonyl sources, transition-metal-catalyzed carbonylative lactonization is most commonly used for *γ*-lactone formation. Iron pentacarbonyl is a cheap, practical surrogate of the carbonyl donor, and it was first applied to convert (amino)polyhydroxylated terminal olefins **122** into the bicyclic lactones **123** by Gracza and colleagues (Figure 34) [136]. In this system, a CO molecule is generated in situ by the assistance of copper(II) chloride and gentle heat, and subsequently participates in the palladium(II) catalysis cycle. Very recently, the same group showed that this protocol could be applicable to a continuous flow reaction in comparable yield with the batch reaction [137].

**Figure 35:**
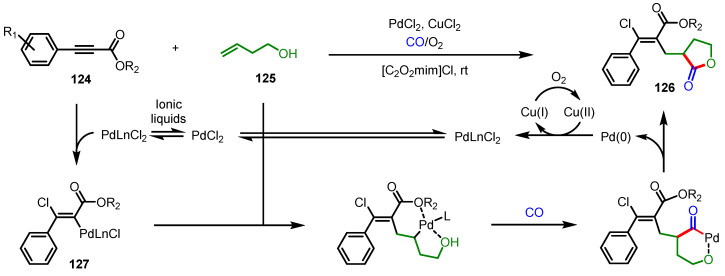
Synthesis of C3-substituted *γ*-butyrolactones via palladium-catalyzed carbonylation cascade in the ionic liquid.

In 2014, Jiang and colleagues reported a unique one-pot-four-step cascade reaction in ionic liquid media by employing a palladium-catalyzed carboxylative annulation to construct highly functionalized *γ*-butyrolactones (Figure 35) [138]. This transformation is initiated from the *trans*-chloropalladation of alkynoates **124**, of which the regioselectivity is governed by electronic factors. Intermediate **127** undergoes carbopalladation with butenol **125**, followed by CO insertion and reductive elimination, yielding C3 functionalized *γ*-lactones **126** bearing a tetrasubstituted olefin unit. The imidazolium type ionic liquids played an important role during the reaction as a ligand of the palladium catalyst and as a chloride source [139]. They further demonstrated the utility of vinyl chloride functionalities in the products by employing them to Suzuki–Miyaura coupling and Negishi coupling.

**Figure 36:**
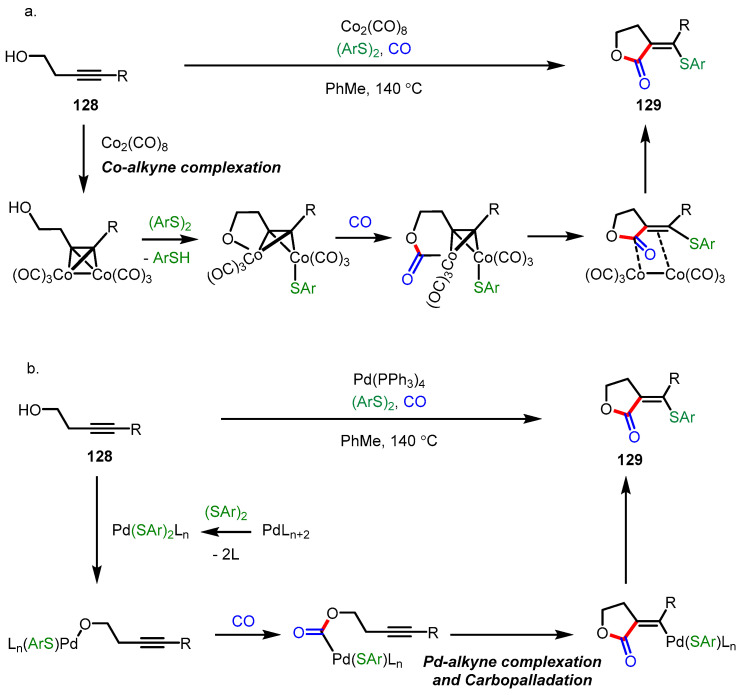
(**a**) Synthesis of thiolated *α*-alkylidene-*γ*-butyrolactones via cobalt-catalyzed carbonylation; (**b**) Synthesis of thiolated *α*-alkylidene-*γ*-butyrolactones via palladium-catalyzed carbonylation.

Organic disulfides, which have been considered as inefficient substrates for transition-metal-catalyzed carbonylative heteroatom addition, were successfully used as counterparts of thiolated *α*-alkylidene-*γ*-butyrolactone synthesis in the presence of dicobalt octacarbonyl or palladium complexes such as Pd(PPh_3_)_4_ and Pd(OAc)_2_ (Figure 36) [140]. A variety of homopropagyl alcohols **128** and aryl disulfides produced the desired thiolated lactones **129** by both catalytic systems with high regio- and stereoselectivity (*cis-*isomer). Mechanistically, despite the difference in the order of metal-alkyne complexation, the presence of a hydroxy group plays a critical role in the regioselectivity of carbonyl insertion in both cases.

**Figure 37:**
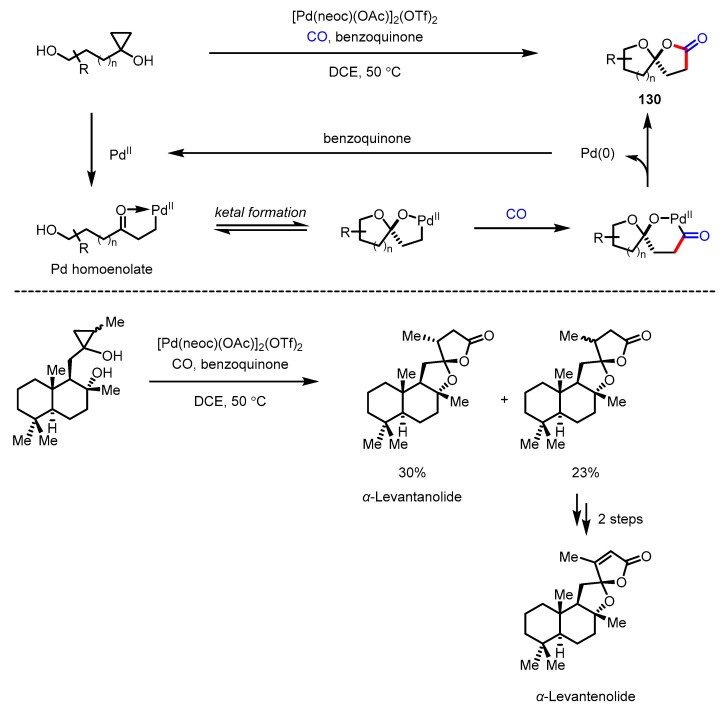
Synthesis of oxaspiro-*γ*-butyrolactones via palladium-catalyzed carbonylative spirolactonization and total synthesis of *α*-levantanolide and *α*-levantenolide.

C-C bonds in cyclopropanols can be easily activated by a transition-metal catalyzed ring-opening process generating metal-homoenolate species, which possess the potential of structural diversification by engaging in C_sp3_-C_sp2_ and C_sp3_-C_sp3_ cross-coupling with various counterparts [141]. Dai and colleagues combined this palladium-catalyzed C-C bond activation reaction with conventional carbonylation, and successfully constructed synthetically challenging oxaspirolactone structure **130** (Figure 37) [142]. The usefulness of this strategy was demonstrated by total syntheses of *α*-levantanolide and *α*-levantenolide in two and four steps, respectively (Figure 37, bottom).

#### 3.5.2. Hydroformylation-Oxidation

**Figure 38:**
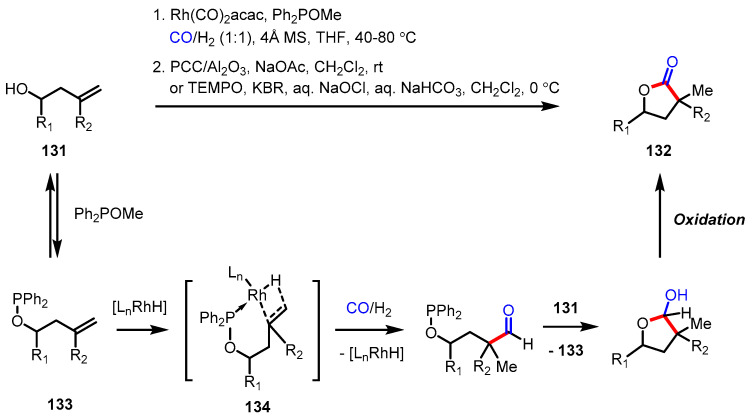
Synthesis of 3,3,5-trisubstituted-*γ*-butyrolactones via rhodium-catalyzed Markovnikov hydroformylation and oxidation.

The hydroformylation of olefins is one of the extensively investigated classes of carbonylation, especially for industrial applications [143]. This reaction is also applicable to *γ*-butyrolactone syntheses by adding a formyl group to hydroxyalkenes and subsequent oxidation of the corresponding lactols. Although the carbonyl insertion step has been known to normally take place in the anti-Markovnikov direction, Breit and colleagues successfully converted 1,1-disubstituted homoallylic alcohols **131** into the desired *γ*-lactones **132** containing quaternary carbon at the *α*-position (Figure 38) [144]. The key to this achievement was the use of a phosphinite as a removable catalyst-directing group. Diphenylphosphinites **133** was formed via transesterification with a catalytic amount of Ph_2_POMe and the resulting phosphinite group-guided approach of the rhodium hydride complex afforded a favorable six-membered cyclic hydrometallation transition state **134**.

**Figure 39:**
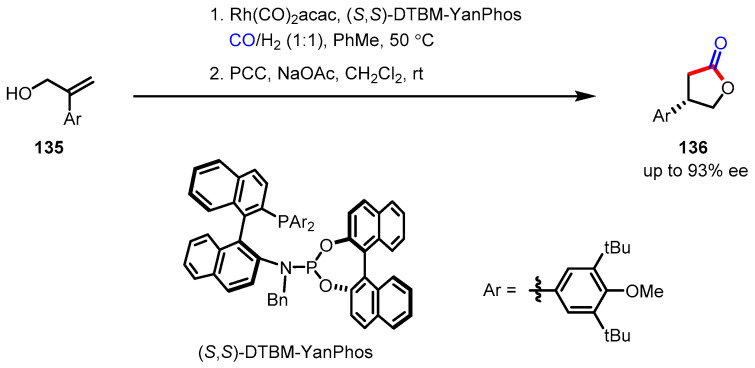
Asymmetric synthesis of 4-substituted *γ*-butyrolactones via rhodium-catalyzed hydroformylation and oxidation.

The enantioselective hydroformylation of 1,1-disubstituted olefins has proven to be unproductive, presumably due to the steric repulsion of an olefin coordination with a metal center [145]. Very recently, Zhang and colleagues addressed this challenge by modifying conventional chiral ligands to more sterically demanding variants (Figure 39) [146]. Under the optimized conditions, the hydroformylation of allylic alcohol **135** occurred following the anti-Markovnikov rule in high ee values, producing the corresponding optically active lactol. The lactol was able to be transformed into not only the desired optically active lactone **136** via PCC oxidation, but also into the tetrahydrofuran derivative via reduction or allylation.

#### 3.5.3. Carboxylation-Lactonization

**Figure 40:**
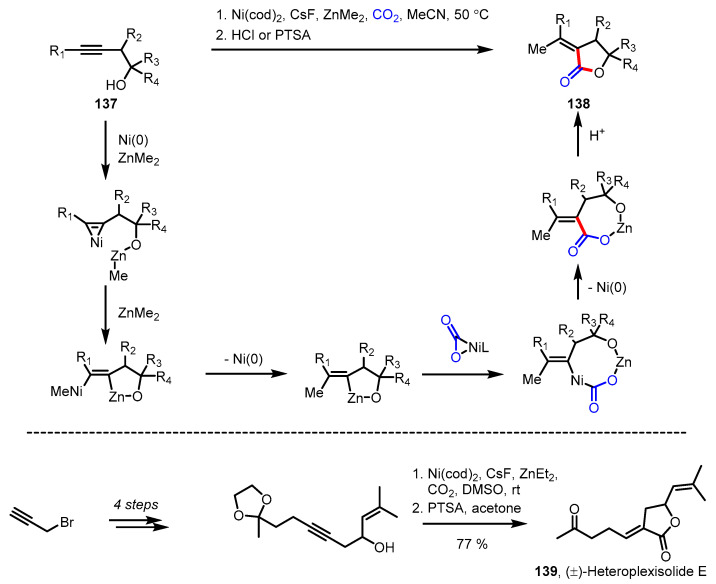
Synthesis of α-alkyledene *γ*-butyrolactones via Ni(0)-catalyzed carboxylation and total synthesis of (±)-heteroplexisolide E.

Carbon dioxide is the most abundant C1 source on earth; thus, harnessing this molecule would be appealing with respect to the development of economical and environmentally friendly synthetic methods. Nevertheless, due to the chemically inert nature of CO_2_ gas, carboxylation (CO_2_ activation) has been less widespread than carbonylation (CO activation). The nickel-catalyzed methyl-carboxylation of homopropagylic alcohols **137** met this demand, affording *α*-alkylidene-*γ*-butyrolactones **138** in a regio- and stereoselective manner (Figure 40) [147]. Ma and colleagues discovered that this catalytic system only required 1 mol % of Ni catalyst for CO_2_ activation and proceeded with broad functional group tolerance. The excellent regioselectivity may derive from the directing effect of the adjacent hydroxy group. The potential of this methodology was illustrated through the first total synthesis of (±)-heteroplexisolide E **139** [148].

### 3.6. Synthesis of γ-Butyrolactones via C3-C4 Bond Formation

#### C-H Insertion

**Figure 41:**
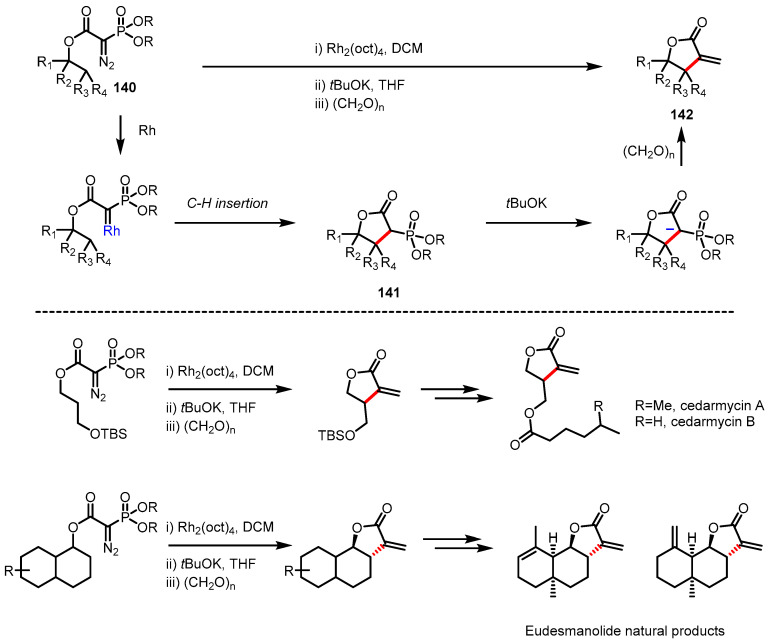
Synthesis of *γ*-butyrolactones and natural products via Rh-catalyzed C-H insertion.

Over the past several decades, Rh-catalyzed intramolecular C-H insertion has been intensively investigated and established as a powerful tool for the construction of structurally diverse cyclic compounds. Unsworth and colleagues reported a one-pot C-H insertion/olefination sequence to afford *α*-alkylidene-*γ*-butyrolactones (Figure 41) [149]. Rh-catalyzed C-H insertion of diazo compound **140** gave *α*-phosphonated *γ*-lactone **141**, which was subsequently converted to *α*-alkylidene-*γ*-lactone **142** via Horner–Wadsworth–Emmons-type olefination. A variety of *γ*-lactones were obtained in a one-pot procedure in useful yields. The versatility of this protocol was demonstrated by the successful synthesis of natural products, cedamycin A, B, and eudesmanolide [150,151].

### 3.7. Synthesis of γ-Butyrolactones via Oxidative C2-O1 Bond Formation

A simple *γ*-butyrolactone is itself a broadly used material [152] as a solvent, extraction agent, and intermediate for polymers, pharmaceutics, herbicides, rubber production, etc. The oxidative lactonization of 1,4-butanediol under an efficient catalytic system has been a dominant industrial process because of its significant advantages [152]. This method does not produce any waste except for reusable hydrogen gas. Additionally, 1,4-butanediol can be obtained from renewable biomass such as glucose [153]. For these reasons, it is not surprising that many researchers have intensively modified this route to be more efficient and environmentally benign than conventional methods. The representative oxidative lactonization conditions recently developed for the synthesis of *γ*-butyrolactones from 1,4-butanediol are summarized in Table 4.


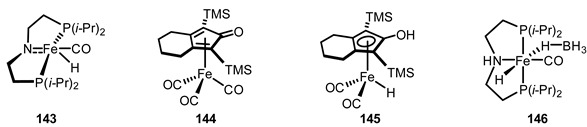
.

## 4. Conclusions

*γ*-Butyrolactones have been broadly studied in drug discovery, resulting in the identification of diverse biologically active small molecules containing *γ*-butyrolactone. Moreover, significant efforts to develop efficient and concise synthetic strategies toward *γ*-butyrolactone moiety have been reported in recent years utilizing readily available starting materials and newly developed reactions. The construction of diverse biologically active natural products and synthetic pharmaceuticals bearing *γ*-butyrolactone are allowed with these novel strategies. This review includes a brief overview of biologically active *γ*-butyrolactones and a summary of the representative synthetic methodologies toward *γ*-butyrolactones developed between 2010 and 2020, which are classified in the seven sections based on the sites of bond formation (Table 5) and described their reaction mechanism and further application in the synthesis of biologically active molecules. This update will help to develop biologically active new *γ*-butyrolactones and to solve hurdles in the synthesis of *γ*-butyrolactone-bearing natural products and pharmaceuticals as well as to develop novel synthetic approaches toward *γ*-butyrolactones.

## Figures and Tables

**Table 1:** Approved drugs containing *γ*-butyrolactone moiety.

Entry	Name	Structure	Target Protein	Disease	Source	Reference
1	Pilocarpine	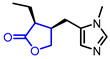	Muscarinic receptor	Xerostomia	Natural	[15]
2	Spironolactone	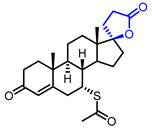	Mineralocorticoid receptor	Heart failure, Hypertension	Synthetic	[16]
3	Eplerenone	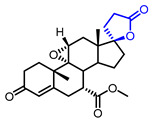	Mineralocorticoid receptor	Heart failure, Hypertension	Synthetic	[17]
4	Drospirenone	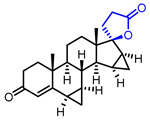	Progesterone receptor	Oral contraceptive	Synthetic	[18]
5	Podofilox	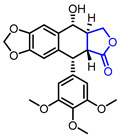	DNA topoisomerase II	Genital warts	Natural	[19]
6	Etoposide	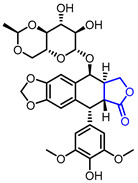	DNA topoisomerase II	Lung cancer, Leukaemia	Synthetic	[20]
7	Teniposide	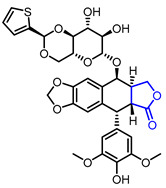	DNA topoisomerase II	Lymphoblastic leukaemia	Synthetic	[21]
8	Vorapaxar	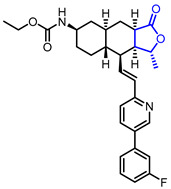	Protease-activated receptor	Thrombotic cardiovascular events	Synthetic	[22]

**Table 2:** Representative biologically active *γ*-butyrolactones.

Entry	Pharmacological Activity	Structure	Name	Bioassay	Source	Reference
1	Anti-inflammation	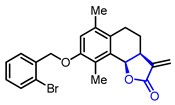	(3a*S*,9b*R*)-8-((2-Bromobenzyl)oxy)-6,9-dimethyl-3-methylene-3,3a,4,5-tetrahydronaphtho[1,2-*b*]furan-2(9b*H*)-one	UbeH5c binding assay (K_d_ = 0.283 μM) Therapeutic effect on adjuvant arthritis rat model	Synthetic	[23,24]
2		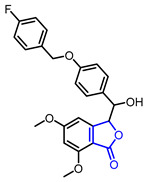	3-((4-((4-Fluorobenzyl)oxy)phenyl)(hydroxy)methyl)-5,7-dimethoxyisobenzofuran-1 (3H)-one	Inhibition rate of NO production at 10 µM (95.23 ± 3.21%) Therapeutic effect on adjuvant arthritis rat model	Synthetic	[25,26]
3		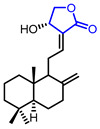	Calcaratarin D	Suppression of NF-κB activation by reducing p65 nuclear translocation Suppression of LPS-induced activation of PI3K/Akt pathway	Natural (*Alpinia calcarata*)	[27]
4		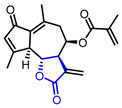	(3aR,4R,9aS,9bR)-6,9-Dimethyl-3-methylene-2,7-dioxo-2,3,3a,4,5,7,9a,9b-octahydroazuleno[4,5-b]furan-4-yl methacrylate	NF-κB inhibition (IC_100_ = 10 μM)	Natural (*Viguiera gardneri*)	[28]
5	Anti-inflammation	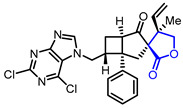	(1*R*,3*R*,4’*R*,5*R*,7*R*)-7-((2,6-Dichloro-7*H*-purin-7-yl)methyl)-4’-methyl-1-phenyl-4’-vinyldihydro-2’*H*-spiro[bicyclo[3.2.0]heptane-3,3’-furan]-2’,4-dione (Biyouyanagin analog)	Inhibition of LPS-induced cytokine production	Synthetic	[29]
6		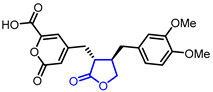	Arctiidilactone	Suppression of LPS-induced NO production	Natural (*Arctium lappa* L.)	[30]
7		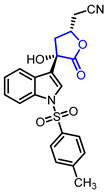	2-((2*S*,4*S*)-4-Hydroxy-5-oxo-4-(1-tosyl-1*H*-indol-3-yl)tetrahydrofuran-2-yl)acetonitrile	COX2 inhibition (IC_50_ < 0.001 uM)	Synthetic	[31]
8		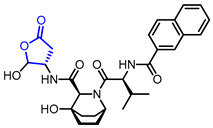	CD10847	Caspase-1 inhibition (IC_50_ = 17 nM)	Synthetic	[32]
9		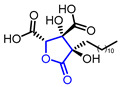	Cinatrin C3	Phospholipase A2 inhibition (IC_50_ = 70 μM)	Natural (*Circinotrichum falcatisporum* RF-641)	[33]
10	Anticancer	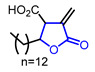	Protolichesterinic acid	Cytotoxicity in HeLa cells	Natural (Lichen metabolites)	[34]
11		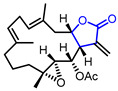	(1a*R*,5*E*,8*E*,10a*S*,13a*S*,14*S*,14a*R*)-1a,5,9-Trimethyl-13-methylene-12-oxo-1a,2,3,4,7,10,10a,12,13,13a,14,14a-dodecahydrooxireno[2’,3’:4,5]cyclotetradeca[1,2-*b*]furan-14-yl acetate	Cytotoxicity in RAW 264.7 cell (IC_50_ = 5.99 μM)	Natural (*Lobophytum* sp.)	[35]
12		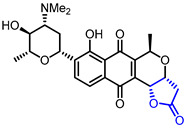	Lactoquinoomycin (Medermycin)	AKT inhibition (IC_50_ = 0.149 μM) Cytotoxicity in MDA468 cells (IC_50_ = 0.05 μM)	Natural (*Streptomyces* K73)	[36,37]
13		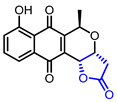	Kalafungin	AKT inhibition (IC_50_ = 0.313 μM) Cytotoxicity in MDA468 cells (IC_50_ = 0.07 μM)	Natural (*Streptomyces tanashiensis*)	[36,38]
14		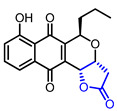	Frenolicin B	AKT inhibition (IC_50_ = 0.198 μM) Cytotoxicity in MDA468 cells (IC_50_ = 0.06 μM)	Natural (*Streptomyces roseofulvus* strain AM-3867)	[36,39]
15	Anticancer	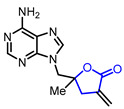	5-((6-Amino-9*H*-purin-9-yl)methyl)-5-methyl-3-methylenedihydrofuran-2(3*H*)-one	Cytotoxicity in L1210 cells (ED_50_ = 0.3 μg/mL)	Synthetic	[40]
16		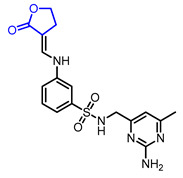	(*E*)-*N*-((2-Amino-6-methylpyrimidin-4-yl)methyl)-3-(((2-oxodihydrofuran-3(2*H*)-ylidene)methyl)amino)benzenesulfonamide	HSP90 binding (Ki = 1.9 μM)	Synthetic	[41]
17	Antibiotic	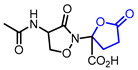	Lactivicin	Inhibition of β-Lactamase in *Proteus vulgaris* (IC_50_ = 2.4 μg/mL)	Natural (Bacteria YK-258 and YK-422)	[42,43]
18		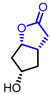	(3a*S*,5*S*,6a*S*)-5-Hydroxyhexahydro-2*H*-cyclopenta[*b*]furan-2-one	Inhibition of β-lactamase in *Klebsiella oxytoca* (IC_50_ = 15 mg/l)	Synthetic	[44]
19	Antibiotic	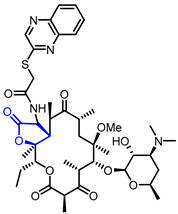	*N*-((3*R*,3a*S*,4*R*,6*R*,8*R*,9*R*,10*R*,12*R*,15*R*,15a*S*)-9-(((2*S*,3*R*,4*S*,6*R*)-4-(Dimethylamino)-3-hydroxy-6-methyltetrahydro-2H-pyran-2-yl)oxy)-15-ethyl-8-methoxy-4,6,8,10,12,15a-hexamethyl-2,5,11,13-tetraoxotetradecahydro-2*H*-furo[2,3-*c*][1]oxacyclotetradecin-3-yl)-2-(quinoxalin-2-ylthio)acetamide	Antibacterial activity against erythromycin-susceptible *Streptococus pyogenes* (MIC = 0.06 μg/mL)	Synthetic	[45]
20		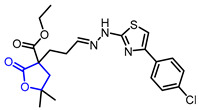	2-Ethoxycarbonyl-2-[2-(3-*p*-chlorophenylthiazol-2- yl)hydrazono]propyl-4,4-dimethylbutanolide	Antibacterial activity against *Staphylococcus aureus*	Synthetic	[46]
21		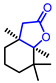	(3a*S*,7a*S*)-3a,7,7,7a-Tetramethylhexahydrobenzofuran-2(3*H*)-one	Antibacterial activity against *Staphylococcus aureus*	Synthetic	[47]
22		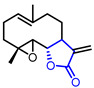	(1a*R*,10a*S*,*Z*)-1a,5-Dimethyl-8-methylene-2,3,6,7,7a,8,10a,10b-octahydrooxireno[2’,3’:9,10]cyclodeca[1,2-*b*]furan-9(1a*H*)-one	Antibacterial activity against MRSA USA300 (MIC = 56.7 μM)	Synthetic	[48]
23		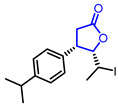	(4*S*,5*S*)-5-((*S*)-1-Iodoethyl)-4-(4-isopropylphenyl)dihydrofuran-2(3*H*)-one	Antimicrobial activity against *Proteus mirabilis* (MIC = 0.25 mg/mL)	Synthetic	[49,50]
24	Antifungal	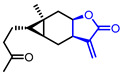	Carabrone	Fungicidal activity against *C. lagenarium* (IC_50_ = 7.10 µg/mL)	Natural (*Carpesium abrotanoides*)	[51]
25		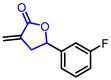	4- (3-Fluorophenyl)-2-methylenebutyrolactone	Fungicidal activity against *C. lagenarium* (IC_50_ = 57.9 µM)	Synthetic	[52]
26		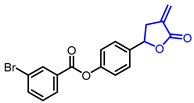	4-[4-(3-Bromobenzoyloxy)phenyl]-2-methylenebutyrolactone	Fungicidal activity against *C. lagenarium* (IC_50_ = 8.76 µM)	Synthetic	[53]
27		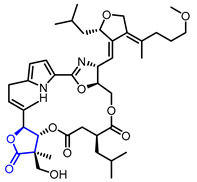	Leupyrrins A1	Fungicidal activity against *M. hiemalis* (MIC = 0.3 µg/mL)	Natural (*Sorangium cellulosum*)	[54]
28	Immunosuppressive	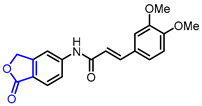	(E)-3-(3,4-Dimethoxyphenyl)-*N*-(1-oxo-1,3-dihydroisobenzofuran-5-yl)acrylamide	Inhibition of T cells proliferation (IC_50_ = 0.029 μM)	Synthetic	[57]
29	Immunosuppressive	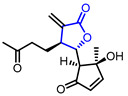	(4S,5S)-5-((1S,2S)-2-Hydroxy-2-methyl-5-oxocyclopent-3-en-1-yl)-3-methylene-4-(3-oxobutyl)dihydrofuran-2(3H)-one	Inhibition of T lymphocyte proliferation (IC_50_ = 1.0 μM)	Natural (*Artemisia argyi*)	[58]
30		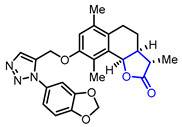	(3*S*,3a*S*,9b*R*)-8-((1-(Benzo[*d*][1,3]dioxol-5-yl)-1*H*-1,2,3-triazol-5-yl)methoxy)-3,6,9-trimethyl-3a,4,5,9b-tetrahydronaphtho[1,2-*b*]furan-2(3*H*)-one (α-Santonin derivative)	Suppression of LPS-induced B-cell proliferation (50% at 10 μM)	Synthetic	[59]
31		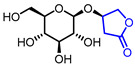	Kinsenoside	VGEFR2 binding Therapeutic effect on autoimmune hepatitis in DCs/Hepa1-6 AIH mouse model	Natural (*Anoectochilus roxburghii*)	[60,61]
32	Neuroprotective	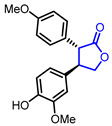	(3*R*,4*R*)-4-(4-Hydroxy-3-methoxyphenyl)-3-(4-methoxyphenyl)dihydrofuran-2(3*H*)-one	Neuroprotective activity in SH-SY5Y cells	Natural (*Cinnamomum* *cassia*)	[62]
33	Neuroprotective	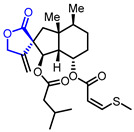	Japonipene C	Neuroprotective activity in SH-SY5Y cells	Natural (*Petasites japonicas*)	[63]
34		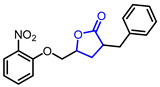	3-Benzyl-5-((2-nitrophenoxy)methyl)dihydrofuran-2(3*H*)-one (3BDO)	PC 12 cell viability assay Alleviation of memory deficits in AβPP/PS1 transgenic mice	Synthetic	[64,65]
35	Antioxidant	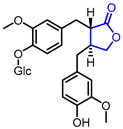	Styraxlignolide E	DPPH Radical-Scavenging Activity (IC_50_ = 194 µM)	Natural (*Styrax japonica*)	[66]
36		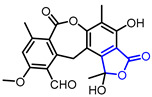	Norstictic acid	Superoxide scavenging Activity (IC_50_ = 580 µM)	Natural (*Usnea articulate*)	[67]
37	Hypoglycemic	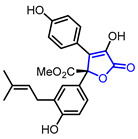	Butyrolactone I	α-Glucosidase inhibition Multiple anti-type 2 diabetic activities in db/db mice	Natural (*Aspergillus terreus*)	[68]
38	Hypoglycemic	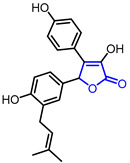	BL-3	PTP1B Inhibitory Assay	Synthetic	[69]

**Table 3:** Radical precursors in photoredox-catalyzed γ-butyrolactone synthesis.

Entry	R	R-RP	PC	Ref
1	Aryl	ArN_2_^+^BF_4_^-^	Ru(bpy)_3_(PF6)_2_	[97]
2	CF_3_	Umemoto’s reagent	Ru(bpy)_3_(PF6)_2_	[98]
3	Alkyl	NHP ester	Ir(ppy)_2_(dtbbpy)PF_6_	[99]

C = photocatalyst, RP = radical precursors.

**Table 4:** Recent reports for *γ*-butyrolactone synthesis from 1,4-butanediol.

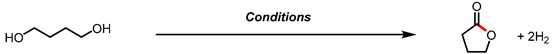			
Entry	Method	Catalyst	Ref
1	Vapor phase reaction	Cu-SiO_2_ nonocomposite	[154]
2	Vapor phase reaction	SiO_2_ supported Cu, Ca, Sr or Br promoter	[155]
3 ^1^	Vapor phase reaction	MgO supported Cu	[156]
4 ^2^	Vapor phase reaction	CaAlO supported Cu	[157]
5 ^3^	Vapor phase reaction	MgO supported Cu, Co_3_O_4_ promoter	[158]
6 ^4^	Vapor phase reaction	MgO supported Cu	[159]
7	Vapor phase reaction	ZrO_2_ supported Cu, La_2_O_3_ promoter	[160]
8 ^5^	Vapor phase reaction	CeO_2_-Al_2_O_3_ supported Cu	[161]
9 ^6^	Continuous flow reaction	AlOx supported Cu nanoparticle	[162]
10	Chemoenzymatic reaction	Type II FMO-E and HLADH	[163]
11	Chemoenzymatic reaction	HLADH	[164]
12	Heterogeneous solution phase reaction	SnO_2_ supported Au	[165]
13	Heterogeneous solution phase reaction	Mn_2_O_3_ supported Au	[166]
15	Homogeneous solution phase reaction	Cu/nitroxyl	[167]
16	Homogeneous solution phase reaction	Fe complex **143**	[168]
17	Homogeneous solution phase reaction	Fe complex **144**	[169]
18	Homogeneous solution phase reaction	Fe complex **145**	[170]
19	Homogeneous solution phase reaction	Fe complex **146**	[171]

^1^ Simultaneous hydrogenation of acetophenone; ^2^ Simultaneous hydrogenation of furfural alcohol; ^3^ Simultaneous hydrogenation of nitrobenzene; ^4^ Simultaneous hydrogenation of *ortho*-chloronitrobenzene.^5^ Simultaneous hydrogenation of benzaldehyde; ^6^ Simultaneous hydrogenolysis of furfural derivatives.

**Table 5:** Summary of synthetic methodologies for the synthesis of *γ*-butyrolactones (2010-2020).

Section	Bond Formation	Reaction	Page
3.1	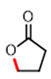	Oxidative lactonization	12
		Halolactonization	14
		Acid-promoted cyclopropane opening	15
		Au-catalyzed oxaallylation	16
		Photoredox-catalyzed lactonization	17
3.2	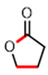	Transition-metal catalyzed C-C bond coupling	18
		NHC-catalyzed C-C bond coupling	20
		Photoredox-catalyzed C-C bond coupling	23
		Miscellsious γ-butyrolactone formation	24
3.3	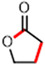	Ruthenium pincer-catalyzed hydrogen autotransfer	27
		Ionic liquid-assisted epoxide opening and lactonization	27
3.4	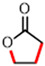	Polar radical crossover cycloaddition (PRCC)	28
		Atom-transfer radical addition (ATRA)	29
		Mn(OAc)_3_-mediated radical lactonization	30
		Copper-catalyzed cyclopropanol ring-opening cross-coupling	30
3.5	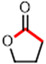	Carbonylative lactonization	31
		Hydroformylation-oxidation	33
		Carboxylation-lactonization	35
3.6	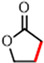	C-H insertion	36
3.7	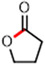	Oxidative C2-O1 bond formation	37

## Data Availability

Not applicable.

## Abbreviations

[C_2_O_2_ mim]Cl	1-carboxymethyl-3-methylimidazolium chloride
Ac	Acetyl
acac	Acetylacetone
Acr	Acridinium
Ar	Aryl
ATRA	Atom-transfer radical addition
Bn	Benzyl
Boc	*tert*-Butyloxycarbonyl
bpy	2,2′- bipyridine
Bu	Butyl
CDI	Carbonyldiimidazole
cod	1,5-Cyclooctadiene
Cp	Cyclopentadienyl
DBU	1,8-Diazabicyclo(5.4.0)undec-7-ene
DCE	1,2-Dichloroethane
dF(CF_3_)ppy	2-(2,4-Difluorophenyl)-5-(trifluoromethyl)pyridine
DFT	Density functional theory
DKR	Dynamic kinetic resolution
Dmim	1,3-Dimethylimidazolium
DMSO	Dimethyl sulfoxide
DPPP	1,3-Bis(diphenylphosphino)propane
dtbbpy	4,4′-Di-tert-butyl-2,2′-bipyridine
EDC	1-Ethyl-3-(3-dimethylaminopropyl)carbodiimide
Et	Ethyl
FMO	Flavin-containing monooxygenase
HAT	Hydrogen atom transfer
HATU	1-[Bis(dimethylamino)methylene]-1H-1,2,3-triazolo[4,5-b]pyridinium3-oxide hexafluorophosphate
Hbim	1-Dutylimidazolium
HLADH	Horse liver alcohol dehydrogenase
HOBt	1-Hydroxybenzotriazole
LED	Light-emitting diode
Me	Methyl
Mes	Mesitylene
MS	Molecular sieve
NBS	*N*-Bromosuccinimide
neoc	Neocuproine
NHC	*N*-heterocyclic carbene
Pc	Phthalocyanine
PCC	Pyridinium chlorochromate
PCR	Peptide coupling reagent
PET	Photoinduced electron transfer
Ph	Phenyl
Phen	Phenanthroline
Pr	Propyl
PRCC	Polar radical crossover cycloaddition
PTSA	*p*-Toluenesulfonic acid
SET	single-electron transfer
TBAF	Tetra-*n*-butylammonium fluoride
TBAP	Tetra-*n*-butylammonium phosphate
TBS	*tert*-Butyldimethylsilyl
TEA	Triethylamine
TEMPO	2,2,6,6-Tetramethylpiperidin-1-yl)oxyl
Tf	Trifluoromethanesulfonyl
TFA	trifluoroacetic acid
THF	tetrahydrofuran
TMBTP	(-)-2,2′,5,5′-tetramethyl-3,3′-bis(diphenylphosphine)-4,4′-bithiophene
TMS	Trimethylsilyl
Ts	*p*-Toluenesulfonyl
